# Predicting brain tumour enhancement from non-contrast MRI with artificial intelligence: a multicohort, retrospective, diagnostic accuracy study

**DOI:** 10.1016/j.lanmit.2026.100012

**Published:** 2026-09

**Authors:** James K Ruffle, Samia Mohinta, Guilherme Pombo, Asthik Biswas, Alan Campbell, Indran Davagnanam, David Doig, Ahmed Hammam, Harpreet Hyare, Farrah Jabeen, Emma Lim, Dermot Mallon, Stephanie Owen, Sophie Wilkinson, Sebastian Brandner, Parashkev Nachev

**Affiliations:** a Department of Translational Neuroscience and Stroke, Queen Square Institute of Neurology, University College London, London, UK; b Lysholm Department of Neuroradiology, National Hospital for Neurology and Neurosurgery, London, UK; c NVIDIA, Reading, UK, UK; d Great Ormond Street Hospital for Children, London, UK; e Imaging Department, Royal National Orthopaedic Hospital, Stanmore, Middlesex, UK; f Department of Radiology, University College Hospitals NHS Foundation Trust, London, UK; g Department of Radiology, Royal Free Hospital, London, UK; h Department of Imaging, Imperial College Healthcare NHS Trust, London, UK; i Department of Brain Sciences, Imperial College London, London, UK; j Division of Neuropathology and Department of Neurodegenerative Disease, Queen Square Institute of Neurology, University College London, London, UK

## Abstract

**Background:**

Brain tumour imaging assessment typically requires both pre-contrast and post-contrast MRI, but gadolinium administration is not always desirable—for example, in patients requiring frequent follow-up, or those with renal impairment or allergy, or those who are of pediatric age [0-18 years]. We aimed to develop and validate a deep learning model to predict brain tumour contrast enhancement from non-contrast MRI sequences alone.

**Methods:**

In this multicohort, retrospective study, we compiled 11 089 brain MRI studies (acquired in 2006–24) from ten datasets across four countries (the UK, the USA, the Netherlands, and Nigeria) and three continents (North America, Europe, and Africa), spanning adult and paediatric populations with no age restriction, and including glioma, meningioma, metastases, and pre-treatment and post-treatment appearances. Three deep learning architectures (nnU-Net, SegResNet, and SwinUNETR) were trained to detect and segment enhancing tumour tissue from T1-weighted (T1w), T2-weighted (T2w), and T2/fluid-attenuated inversion recovery (FLAIR) sequences alone. Performance was assessed in a held-out test set of 1109 studies, with patient-level enhancement detection as the primary endpoint and voxel-level Dice score as the secondary endpoint. Eleven expert radiologists performed the same novel task on a 564-case subset, each reviewing 100 cases while being blinded to clinical history, previous imaging, and referral indication.

**Findings:**

The best performing model, nnU-Net, reached a balanced accuracy of 83·0% (95% CI 79·1–87·2; sensitivity 91·5% [89·9–93·1], specificity 74·4% [66·7–82·5]) for enhancement detection, with R^2^=0·859 for enhancement-volume prediction. Among the enhancing cases, 762 (77%) of 992 reached a Dice score of greater than or equal to 0·3, 670 (68%) of 992 had a Dice score of greater than or equal to 0·5, and 498 (50%) of 992 showed a Dice score of greater than or equal to 0·7. In the 564 matched cases, the radiologists’ majority-vote performance was lower, with a balanced accuracy of 71·7% (sensitivity 77·6%, specificity 65·8%) under the same blinded conditions. Patient-level enhancement detection was generally consistent across pathologies (proportion of patients reaching voxel-level Dice ≥0·3: meningioma, 93 [93%] of 100; presurgical glioma, 526 [76%] of 691; metastases, 52 [74%] of 70; post-treatment glioma, 82 [74%] of 111), but was lower for paediatric cases (nine [45%] of 20).

**Interpretation:**

Deep learning can identify contrast-enhancing brain tumours from non-contrast MRI. These models show promise as a triage or decision-support aid—for example, flagging studies in which post-contrast enhancement is likely so that contrast can be added to a non-contrast-only protocol—and could reduce gadolinium dependence in neuro-oncology imaging. Future studies should optimise these models through close collaboration with expert radiologists.

**Funding:**

European Society of Radiology; European Institute for Biomedical Imaging Research (EIBIR); Medical Research Council; Wellcome Trust; UCLH NIHR Biomedical Research Centre.

## Introduction

Imaging diagnosis and surveillance play a key role in neuro-oncological care across adult and paediatric populations.^1^ The presence or absence of contrast enhancement is often crucial to clinical decision making. However, contrast agents cannot always be administered because of contraindications such as allergies or renal impairment, or patient refusal. Imaging could also be affected by artefacts, making radiological interpretation difficult. Furthermore, minimising repeated gadolinium use is often desirable, especially in paediatric patients, although the risk of inadequate tumour characterisation is typically perceived to outweigh the risks associated with repeated gadolinium administration. In addition to known risks, such as the development of nephrogenic systemic fibrosis, the long-term effects of exposure to gadolinium and its deposition in the body remain unknown.^2^

**Research in context**

**Evidence before this study**

We searched PubMed, Embase, and Web of Science from database inception to April 1, 2026, using combinations of (“brain MRI” OR “brain tumour” OR “glioma”) AND (“deep learning” OR “convolutional neural network” OR “U-Net” OR “transformer”) AND (“contrast enhancement” OR “gadolinium” OR “virtual contrast” OR “synthetic contrast”). No language restrictions were applied. Reference lists of retrieved studies were additionally screened. Previous research showed the feasibility of predicting or synthesising contrast enhancement from non-contrast brain MRI using deep learning, but the available evidence was confined to single-institution, adult, predominantly glioma cohorts of modest size, with most studies enrolling tens to hundreds of patients and only rarely extending to larger, multi-institutional cohorts. Reported performance metrics were heterogeneous across studies, spanning synthetic-image quality indices (structural similarity index and peak signal-to-noise ratio) and enhancing-tumour Dice coefficients, with no consistent patient-level detection benchmarks being reported. Previous studies on gadolinium dose reduction have shown that deep learning could preserve image quality at a quarter to a tenth of the standard contrast-agent dose, although such work still requires gadolinium administration.

**Added value of this study**

To the best of our knowledge, this is the largest and most heterogeneous evaluation of contrast-enhancement prediction from non-contrast brain MRI to date, and the first to directly benchmark deep learning against a blinded expert-radiologist panel on the same non-contrast task, to include a paediatric cohort, and to report performance stratified by demographic and geographic subgroup with a formal equity assessment. Additionally, we quantified the drivers of failure (of the 260 cases with poor enhancement delineation [Dice <0·3]: 105 involved lesions <0·5 cm³ [40·4%], 100 involved atypical enhancement [38·5%], 30 were false positives [11·5%], 17 involved post-treatment change [6·5%], and eight were paediatric cases [3·1%]) and provided a descriptive characterisation of predictive entropy. The approach proved feasible at scale across ten international cohorts and five pathology types, but was weakest in the paediatric population, establishing both the promise of this approach and its current limits.

**Implications of all the available evidence**

Deep learning prediction of contrast enhancement from non-contrast brain MRI achieved higher balanced accuracy than expert radiologists on this unfamiliar non-contrast task. Although the model’s performance is currently insufficient to replace contrast-enhanced MRI acquisitions entirely—particularly in the paediatric population, where the model showed reduced accuracy—these findings suggest that imaging features previously only identifiable from post-contrast imaging can be derived from non-contrast imaging alone using a trained artificial intelligence model. Used as a triage or decision-support aid, such a model could flag imaging studies in which post-contrast enhancement is likely, prompting the addition of contrast to a non-contrast-only study and thereby helping patients avoid a second hospital visit and minimising delays to patient care; patients whose studies are predicted to be enhancement-free might, with appropriate caution, proceed without the use of gadolinium. In combination with previous single-institution literature, these findings are consistent with growing evidence supporting measures to reduce gadolinium dependence in imaging assessments, including oncology imaging.

Beyond the individual patient, the widespread use of gadolinium-based contrast agents in medical imaging has broader environmental implications. Although only small amounts are used in each study, cumulative global use results in substantial amounts (on the order of many thousands of litres) of gadolinium being administered, secreted, or disposed of each year.^3^ Gadolinium has been detected in sewage, surface, and drinking water, water sources distant from MRI facilities, and even fast-food soft drinks. Given the uncertainty surrounding the long-term effects of exposure, even at low doses, the problem has prompted worldwide research efforts to reduce or even eliminate gadolinium use.

One possible solution to reducing dependence on contrast agents is to use artificial intelligence to predict the presence of enhancement from non-contrast imaging alone. We have recently shown a proof of concept that deep learning models can segment enhancing tumour tissue in glioma without contrast-enhanced imaging.^4^ Similar approaches have also been investigated by others in both the brain and other anatomical regions, to either reduce gadolinium dosage^5^^,^^6^ or eliminate its use entirely for lesion detection or image reconstruction.^6^^,^^7^ Collectively, these studies provide strong evidence that complex models can leverage non-contrast MRI sequence information to derive post-contrast information from non-contrast scans.

Here, we prototype an enhanced intracranial disease prediction system using only non-contrast MRI data from the largest known neuro-oncology imaging cohort. We combine state-of-the-art deep learning with large-scale, unselected neuro-oncology data. In addition to its scale, our methodological contributions include (1) a systematic comparison of convolutional neural network and transformer-based architectures; (2) descriptive characterisation of predictive entropy; (3) a comprehensive equity assessment across demographic, clinical, and country-of-origin subgroups; and (4) direct benchmarking against expert human radiologist performance.

## Methods

### Study design and patients

In this multicohort, retrospective study, we combined in-house and international open-source brain tumour data from 10 datasets to study a final dataset of 11 089 imaging sessions from 8548 unique patients diagnosed with a brain tumour (figure 1; appendix p 1). This cohort was as maximally heterogeneous and inclusive as possible, encompassing adult and paediatric patients, pre- and post-treatment imaging, multiple neuro-oncological pathologies, including glioma, meningioma, and brain metastases, and numerous global study sites (UK, USA, the Netherlands, and Nigeria; appendix pp 1, 6, 8). Imaging was acquired between 2006 and 2024 at multiple field strengths (1·5T and 3T) across multiple MRI manufacturers.

**Figure 1:**
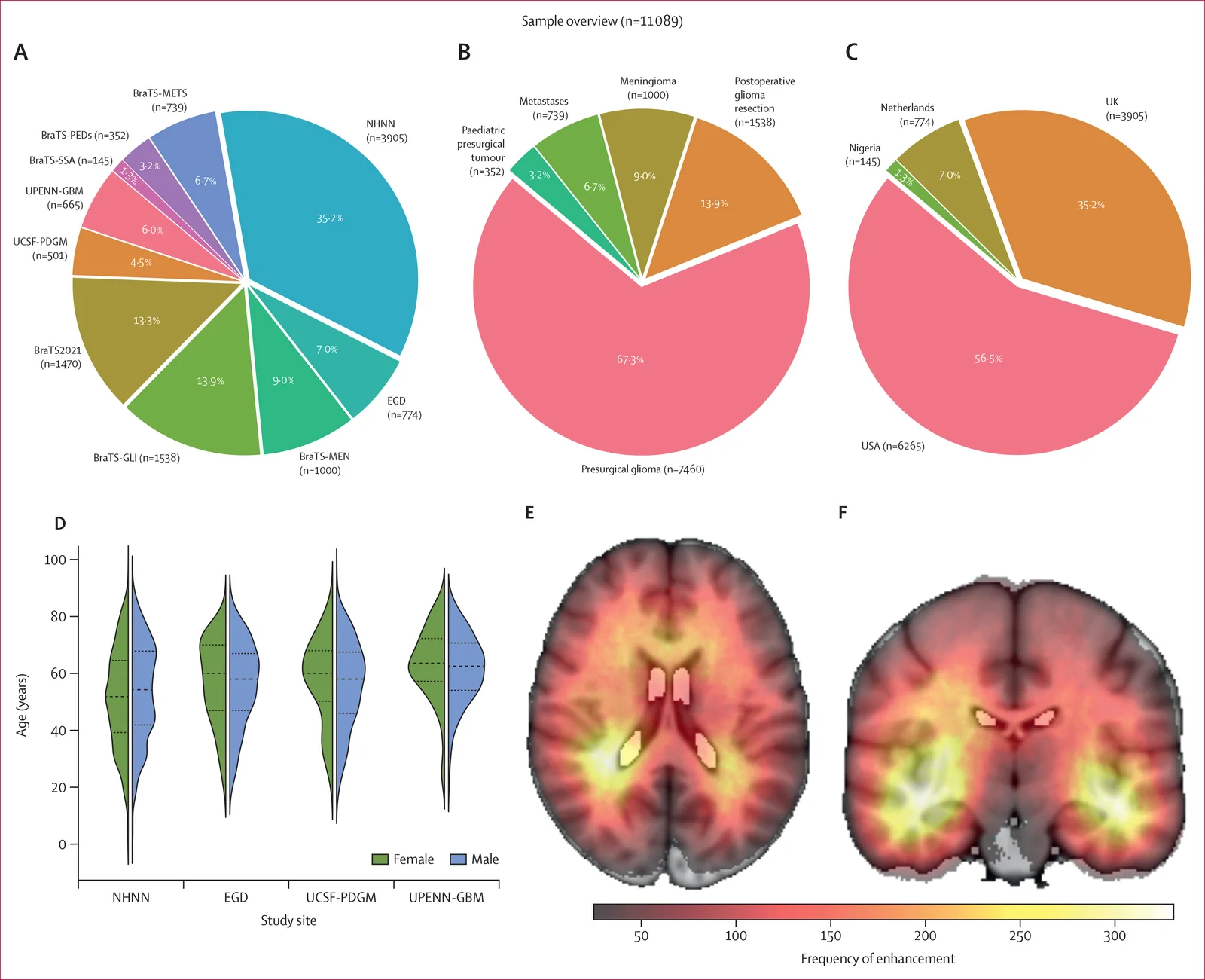
Cohort overview (A) Cohort distribution across the ten different datasets. (B) Distribution of pathology types. (C) Distribution by country of sample origin. (D) Age and sex distribution of the cohort, where available (5187 [46·8%] of 11089 imaging sessions). The lines represent the median, 25th and 75th percentiles. (E–F) Axial and coronal maps of enhancing lesion distribution, with warmer colours indicating a higher frequency of enhancement occurrence at a given anatomical location. BraTS=Brain Tumor Segmentation Challenge. BraTS-GLI=Post-treatment Glioma. BraTS-MEN=Meningioma. BraTS-METS=Metastases. BraTS-PEDs=Paediatric. BraTS-SSA=Sub-Saharan Africa. EGD=Erasmus Glioma Database. NHNN=National Hospital for Neurology and Neurosurgery. UCSF-PDGM=University of California San Francisco Preoperative Diffuse Glioma Dataset. UPENN-GBM=University of Pennsylvania Glioblastoma Dataset.

Eligibility criteria were deliberately broad. Any patient with a brain tumour MRI study available in the contributing datasets was eligible, with no age restrictions (spanning paediatric to adult patients) and no exclusions based on histology, molecular profile, performance status, or comorbidity. As this retrospective analysis used existing imaging data, trial-specific eligibility criteria—including formal disease-response evaluation, performance status, estimated life expectancy, eligibility laboratory tests, and previous-treatment or washout requirements—did not apply. No patients were excluded on eligibility grounds. The cohort therefore comprised all available brain-tumour MRI studies. Cases not meeting image-quality control (inter-sequence motion or registration error), together with cases flagged as cross-dataset duplicates (r>0·95), were removed at preprocessing. These counts were not separately recorded. As the public cohorts were pre-curated by their providers, per-dataset screening counts were not separately re-logged in this secondary analysis.

Patients and members of the public were involved in discussing the research question and study design, but not the choice of outcome measures, the conduct of the study, the analysis or interpretation of the data, or the writing of the report. The local ethics committee at University College London approved the study. We received approval for analysis of local irrevocably anonymised data collected during routine clinical care without individual patient consent (approval reference 07/H0707/152). All external datasets also received ethical approval, and their consent procedures are described within the source datasets.^8^, ^9^, ^10^, ^11^, ^12^, ^13^, ^14^, ^15^, ^16^

### Model development

We trained a series of fully volumetric, multimodal, three-dimensional (3D) deep learning models to segment enhancing tumours from non-contrast imaging sequences (figure 2). Models received ground-truth labels for background (ie, non-brain), normal brain parenchyma, non-enhancing abnormal tissue, and enhancing neuro-oncological disease, which was the primary target of interest. The derivation of the ground-truth labels is further described in the appendix (p 2). Notably, the enhanced tumour component was always derived from complete datasets, including post-contrast T1-weighted (T1w) sequences.

**Figure 2:**
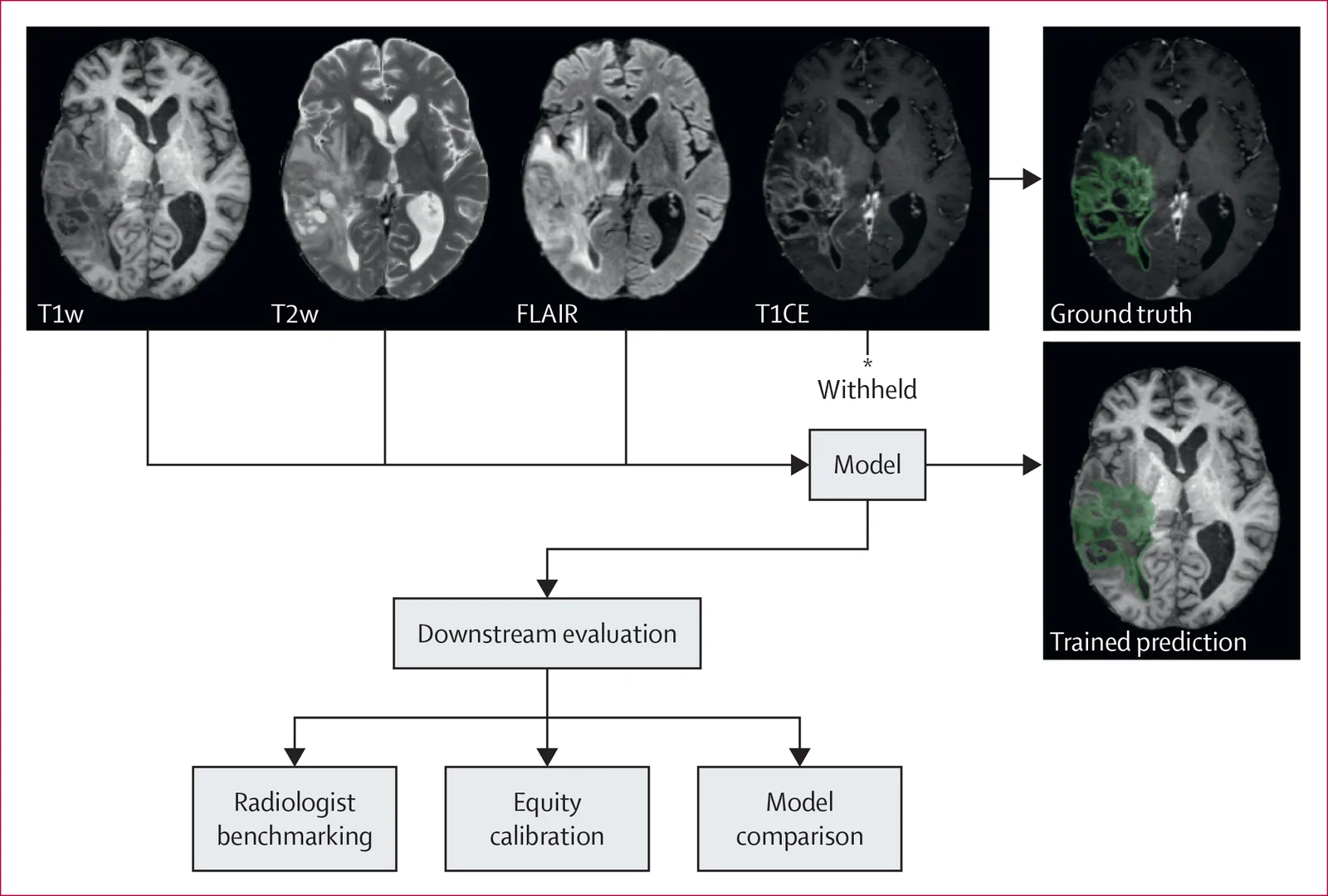
Study schematic The model was trained to predict enhancing intracranial disease using only T1w, T2w, and T2/FLAIR-weighted imaging. Ground-truth labels were generated from complete imaging datasets containing all available sequences (T1w, T2w, T2/FLAIR, and T1CE), with T1CE serving as the reference sequence for enhancement delineation. Model inputs consisted exclusively of non-contrast sequences. Downstream model evaluation comprised conventional model comparison, radiologist benchmarking, and equity assessment. FLAIR=fluid-attenuated inversion recovery. T1CE=post-contrast T1. T1w=T1-weighted. T2w=T2-weighted.

However, model inputs consisted only of non-contrast sequences, specifically T1w, T2-weighted (T2w), and T2/ fluid-attenuated inversion recovery (FLAIR)-weighted MRI sequences. We performed a randomised patient-level train-test split, allocating 90% of patients to training and 10% to model testing, with no patient overlap between datasets. No formal a priori sample-size calculation was performed; instead, we included all consecutively eligible imaging sessions from the contributing cohorts over the study period, yielding a training and test set considerably larger than comparable prediction-model studies in neuro-oncological imaging. We locked the test set until model development was complete. Site and scanner identifiers were not provided as model inputs, and we applied no site-level or scanner-level intensity harmonisation. Consequently, the model learned from acquisition heterogeneity rather than being shielded from it (appendix p 2). Trained model architectures were nnU-Net (v2),^17^ SegResNet,^18^ and SwinUNETR^19^ (appendix pp 3–5).

### Evaluation

#### Performance metrics

We evaluated the performance of trained segmentation models on a held-out test set using both voxel-level and patient-level metrics. Voxel-level metrics, computed in 3D across the full volumetric prediction and ground truth for each imaging session, included the Dice coefficient, balanced accuracy, precision, recall, and F1 score. We also derived bootstrapped patient-wise metrics (1000 iterations) for the overall classification of whether a study contained any pathologically enhancing tissue, quantified by balanced accuracy, macro-averaged precision, recall, and F1 score. At the patient level, we classified a case as enhancement-detected if the model correctly predicted any enhancing voxels after thresholding the softmax probability map at 0·5. For clinical interpretability of segmentation quality, we further refined patient and voxel-level detection success thresholds as follows: acceptable detection (Dice ≥0·3); good detection (Dice ≥0·5); and excellent detection (Dice ≥0·7). Spatial predictions with Dice values below 0·3 were considered clinically unreliable and were used as a heuristic safeguard against excessive model sensitivity and high false-positive rates for enhancement detection. We included both voxel-wise metrics and patient-level enhancement detection metrics to encompass both the conventional statistical evaluations undertaken in computer vision research (which typically operate at the voxel level) and those in clinical research (which typically operate at the patient level).

#### Equity assessment

We quantified the epistemic equity of the model,^20^ defined as fairness in performance across patient subgroups, ensuring equitable diagnostic utility regardless of patient characteristics. We assessed variation in fidelity across patient attributes as a function of the model’s performance, considering the cohort site, underlying pathology, country of sample origin, patient age and sex (where available),^21^ and the lesion size and morphology (appendix pp 4–5). In accordance with the Sex and Gender Equity in Research guidelines, information on sex was obtained directly from the source datasets and denotes the biological sex rather than self-reported gender. Race and ethnicity data were unavailable in all source datasets and therefore could not be evaluated as equity attributes.

#### Comparison with radiologists

We conducted an assessment in which expert radiologists predicted whether a lesion would enhance after contrast administration, using only the unenhanced images. We randomly allocated 100 patients from the held-out test set to each of 11 individual experienced radiologists. The 11 radiologists were board-certified specialists (post-Fellowship of the Royal College of Radiologists and post-Certificate of Completion of Training) with either formal neuroradiology subspecialty training or extensive neuro-oncological imaging experience (median years in practice: 11·3 [IQR 7·5–14·5]). All reported brain tumour MRI as part of routine clinical practice across six large London-based hospitals. Cases were randomly sampled from the held-out test set, with stratification to ensure that each radiologist reviewed 50 cases with an enhancing abnormality and 50 without. Each radiologist reviewed exactly 100 cases, generating 1100 reader case evaluations. Random sampling yielded 564 unique imaging sessions from the 1109-study test set, of which 447 contained an enhancing tumour and 117 did not. Some imaging sessions were reviewed by multiple readers, whereas others were reviewed by a single reader. Radiologists reviewed anonymised imaging using the open-source software ITKSnap on a Linux workstation with a 4K monitor. No time limit was imposed. Readers were informed that approximately half of the cases would contain enhancing lesions for statistical purposes but received no additional clinical information. Cases were presented in a randomised order to minimise recall bias. Each radiologist sequentially reviewed the unenhanced MRI images (T1w, T2w, and T2/FLAIR-weighted) from each case and answered the question: “Do you think there will be an enhancing abnormality in this case?” We then statistically compared our model's predictions with those of the radiologist, with both evaluated against the ground truth.

### Analytic compliance

We performed and reported all analyses in accordance with the TRIPOD-AI and CLAIM guidelines. Classical statistical tests included logistic regression, one-way ANOVA, t-tests, Levene's test for equality of variances, and McNemar's exact test. We calculated patient-level performance metrics (balanced accuracy, sensitivity, specificity, precision, and F1) and corresponding 95% CIs using patient-wise bootstrap resampling (1000 resamples). We set a fixed random seed for all analyses. Voxel-level Dice coefficients were compared across architectures using the paired Wilcoxon signed-rank test on per-case Dice, and patient-level detection metrics were compared using a paired case-level bootstrap of differences between architectures (10 000 resamples). The model was compared with the expert-radiologist majority vote for the matched cases using McNemar's exact test. Positive and negative predictive values and clinical utility estimates (contrast administrations potentially avoided and enhancing tumours potentially missed per 1000 scans) were derived from sensitivity and specificity using Bayes' theorem across varying levels of disease prevalence. Inter-rater agreement for the multi-rater subset of the radiologist-reviewed cases was summarised using Cohen's kappa across all reader-pair combinations. Statistical tests were corrected for multiple comparisons using Bonferroni adjustment, with correction applied within each family of comparisons and corrected p values reported throughout.

All p values were two-sided and were reported as exact values where p was greater than or equal to 0·0001, and p less than 0·0001 otherwise. Analysis code was cross-checked for correctness with assistance from Claude Code (Anthropic).

### Role of the funding source

The funders of the study had no role in study design, data collection, data analysis, data interpretation, or writing of the report.

## Results

The study cohort included 11 089 imaging sessions, with 9980 allocated to the training dataset and 1109 assigned to model testing. Patient age was available for 5187 imaging sessions (46·8%), with a mean of 55 years (SD 16·44). Patient sex was available for 5651 imaging sessions (51·0%); among these, 3061 (54·2%) were male patients and 2590 (45·8%) were female patients. No significant differences in patient age (p=0·76), sex (p=0·38), or site distribution (all p>0·05) were noted between the training and test datasets (appendix p 6).

Of the three deep learning architectures trained and evaluated—nnU-Net, SegResNet, and SwinUNETR—nnU-Net showed the strongest fidelity. For patient-level lesion-detection metrics, nnU-Net reached the highest balanced accuracy (0·830; 95% CI 0·791–0·872), exceeding both comparators on all performance metrics, and the highest voxel-level enhancing tumour Dice (paired Wilcoxon signed-rank test on per-case Dice: nnU-Net *vs* SegResNet, p<0·0001; nnU-Net *vs* SwinUNETR, p<0·0001; appendix pp 9–10). nnU-Net was therefore selected as the primary model for all subsequent analyses. Figure 3 shows sample patients from all imaging cohorts.

**Figure 3:**
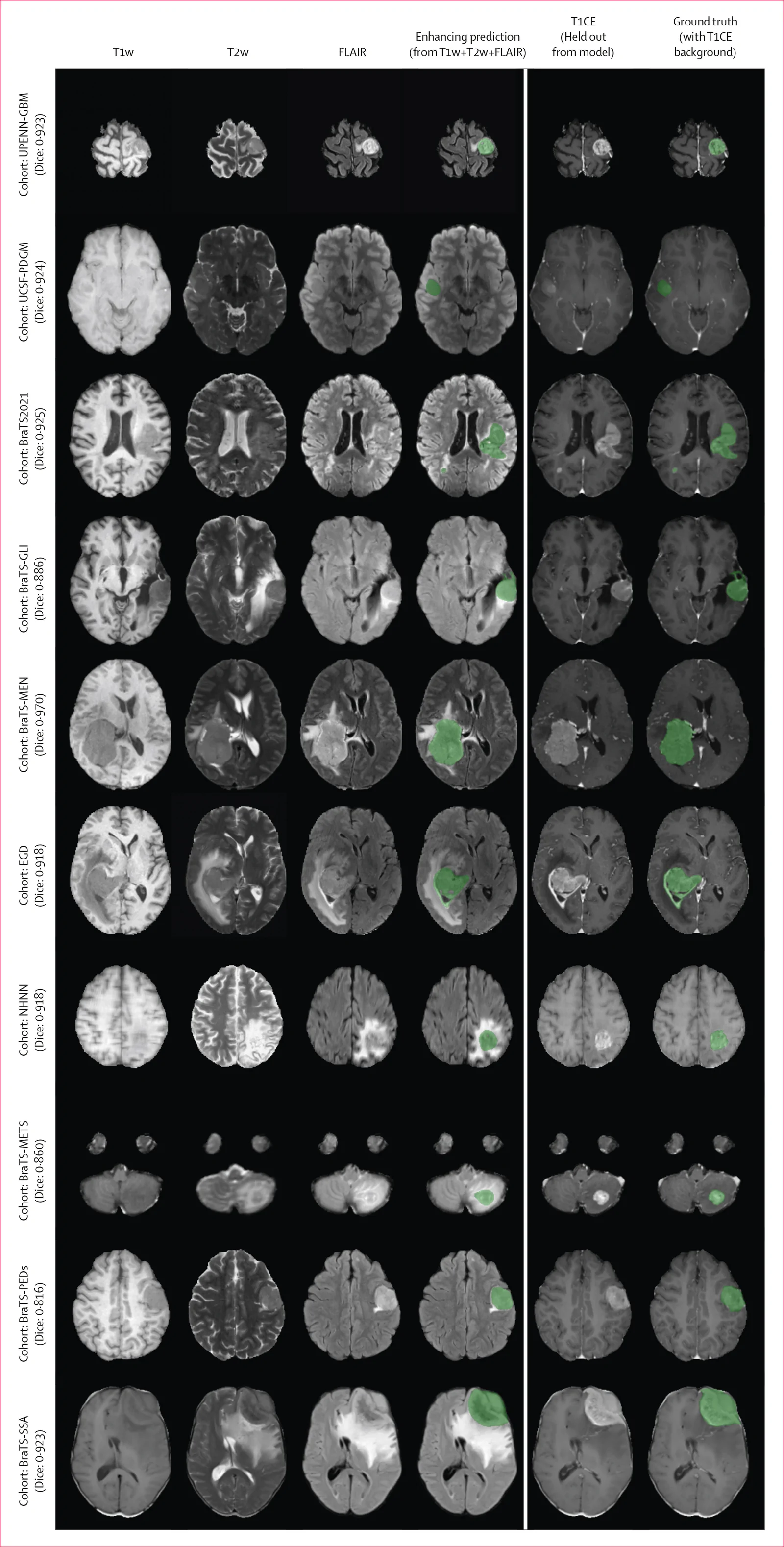
Enhancement prediction from non-contrast sequences in test-set cases Representative test-set cases from each cohort demonstrating model performance. Each row shows a different case with six columns: T1w, T2w, T2/FLAIR-weighted imaging, model-predicted enhancing tumour derived from the unenhanced T1w, T2w, and FLAIR images (green overlay on T2/FLAIR), T1CE (T1 contrast-enhanced, not provided as model input), and the ground-truth enhancing tumour segmentation (overlay on T1CE). BraTS=Brain Tumor Segmentation Challenge. BraTS-GLI=Post-treatment Glioma. BraTS-MEN=Meningioma. BraTS-METS=Metastases. BraTS-PEDs=Paediatric. BraTS-SSA=Sub-Saharan Africa. EGD=Erasmus Glioma Database. FLAIR=fluid-attenuated inversion recovery. NHNN=National Hospital for Neurology and Neurosurgery. T1CE=post-contrast T1. T1w=T1-weighted. T2w=T2-weighted. UCSF-PDGM=University of California San Francisco Preoperative Diffuse Glioma Dataset. UPENN-GBM=University of Pennsylvania Glioblastoma Dataset.

The radiologists, who were not specifically trained for this novel enhancement-prediction task, had a mean balanced accuracy of 0·698 ± 0·072 across 11 readers (bootstrapped aggregate across all 1100 blind reads; 95% CI 0·671–0·725), which was lower than the model's performance. When these 1100 reads were aggregated into a per-case majority vote across the 564 unique cases (appendix p 11), the radiologist panel’s balanced accuracy rose to 0·717, reflecting an incremental improvement from multi-reader consensus. Of the 564 matched cases, the model and radiologists agreed on 421 (both correct in 390 and both incorrect in 31); of the 143 cases on which they disagreed, the model was correct in 109 cases compared with 34 cases for the radiologists (McNemar's exact two-sided test, p<0·0001). Overall, the model was correct in 499 (88% accuracy) of 564 cases, compared with 424 (75%) for the radiologist majority; the corresponding balanced accuracies were 0·833 (95% CI 0·791–0·872) and 0·717 (95% CI 0·670–0·763). Of the 447 enhancing cases in this subset, the radiologists did not correctly identify 100, of which the model correctly identified 79 (79%). Conversely, the model did not correctly identify 35, of which the radiologists correctly identified 14 (40%). Agreement between readers on the multi-rater cases was fair (Cohen's κ=0·36; observed agreement 68%), consistent with the inherent difficulty of predicting enhancement from non-contrast imaging alone. Figure 4 shows sample patient imaging sessions incorrectly classified by radiologists but correctly identified by the model.

**Figure 4:**
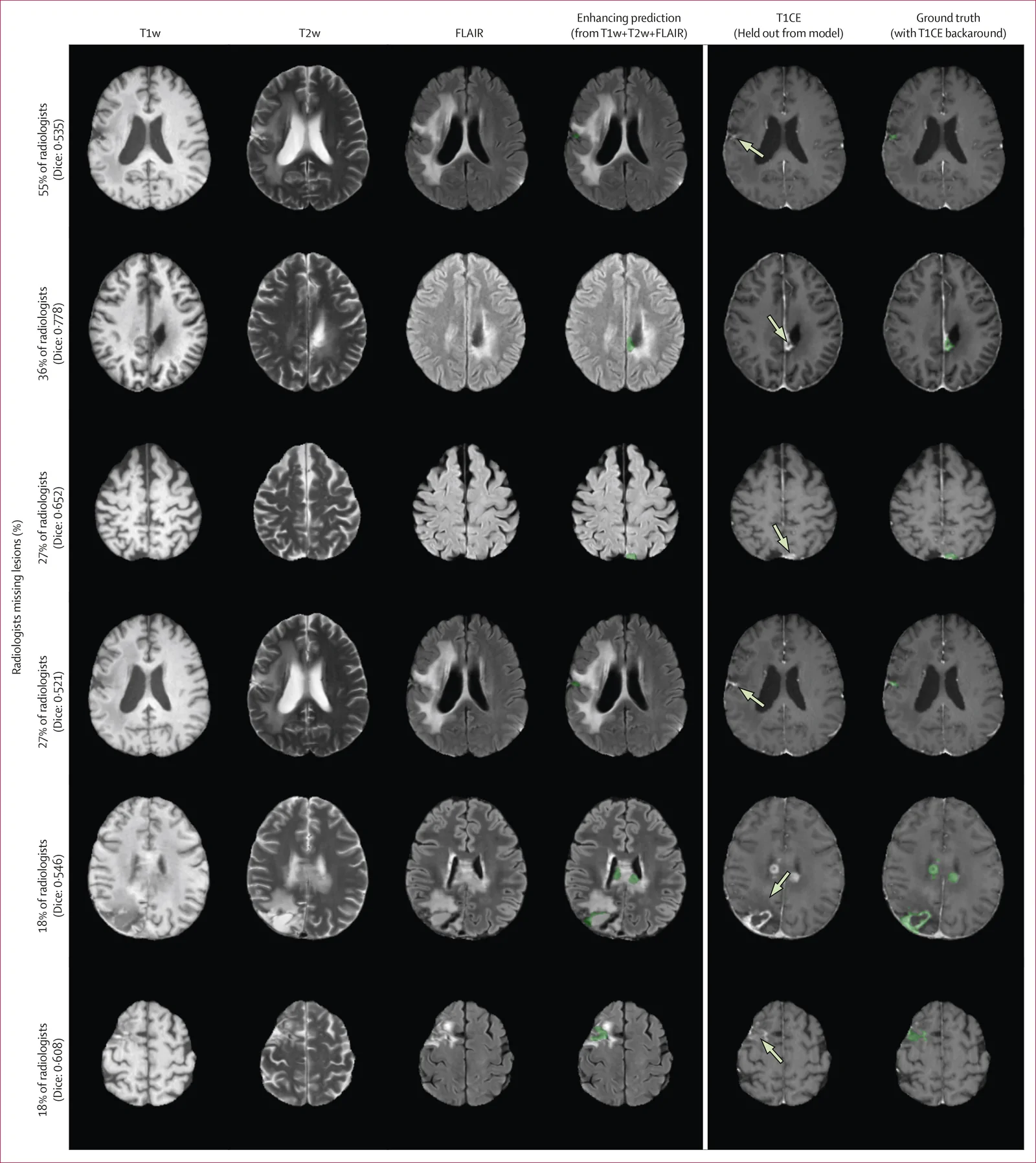
Test-set samples of lesions missed by radiologists Representative test-set cases that radiologists incorrectly predicted to show no post-contrast enhancement when reviewing the non-contrast imaging. Each row shows a different case with six columns: T1w, T2w, FLAIR, model prediction of enhancing tumour from T1w, T2w, and FLAIR images (green overlay on FLAIR), T1CE (T1 contrast-enhanced, not provided as model input), and the ground-truth enhancing tumour segmentation (overlay on T1CE). The reference T1CE images show that model predictions correspond to true contrast-enhancement patterns. Percentages on the left axis indicate the proportion of radiologists who predicted that the lesion would not enhance. Dice scores for the model's prediction on these missed lesions are provided in parentheses. FLAIR=fluid-attenuated inversion recovery. T1CE=post-contrast T1. T1w=T1-weighted. T2w=T2-weighted.

Patient-level enhancement-detection performance, quantified by balanced accuracy, remained robust across the 1109 test-set cases and all datasets, pathologies, countries, patient ages, and sexes, with balanced accuracy ranging from 0·739 to 1·000 (figure 5; appendix pp 12–14). Performance was slightly lower for post-treatment glioma resection cases (balanced accuracy 0·825; figure 5) and paediatric presurgical tumours (balanced accuracy 0·739, 95% CI 0·555–0·900). Detection performance was also lower in our internal National Hospital for Neurology and Neurosurgery (NHNN) cohort (0·790, 0·717–0·856). No significant difference in detection performance was noted between the male (0·816, 0·721–0·894) and female (0·830, 0·738–0·909) patients. Performance stratified by patient age is shown in figure 5; the balanced accuracy was numerically lower in the youngest patients (under-30 0·765 [95% CI 0·577–0·906] *vs* ≥30 0·833 [0·764–0·893]; appendix p 14), with overlapping confidence intervals (n=565, recoverable for 50·9% of studies as the adult Brain Tumor Segmentation Challenge (BraTS) cohorts are age-stripped at source; figure 5).

**Figure 5:**
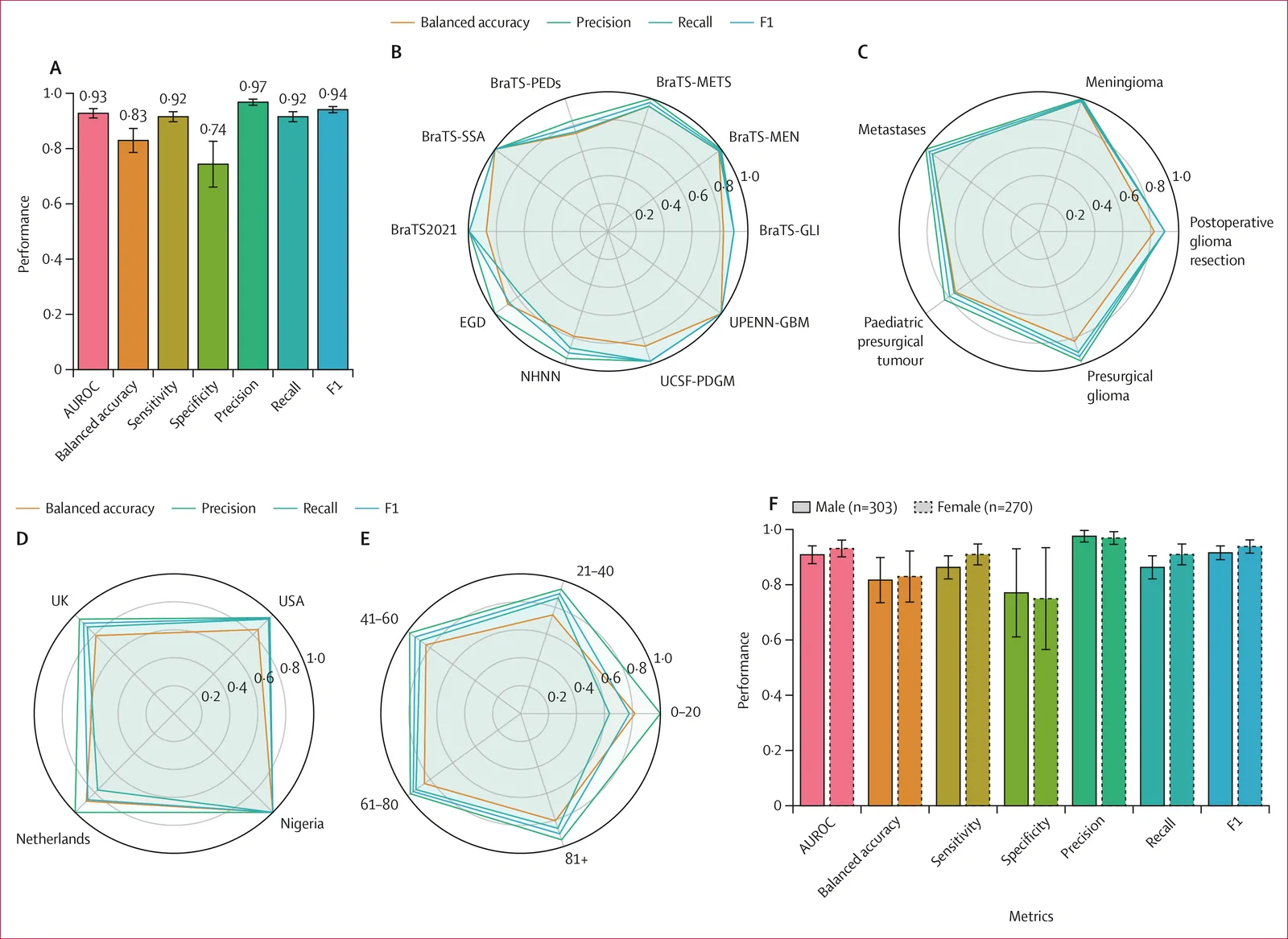
Brain tumour enhancement detection performance Patient-level detection of enhancing tumour from non-contrast sequences in the overall cohort (A), stratified by dataset (B), pathologies (C), country of sample origin (D), age (available for 50·9%) (E), and sex (available for 51·7%) (F). Error bars in panels (A) and (F) represent bootstrapped 95% confidence intervals. AUROC=area under the receiver operating characteristic curve. Balanced Acc=balanced accuracy. BraTS=Brain Tumor Segmentation Challenge. BraTS-GLI=Post-treatment Glioma. BraTS-MEN=Meningioma. BraTS-METS=Metastases. BraTS-PEDs=Paediatric. BraTS-SSA=Sub-Saharan Africa. EGD=Erasmus Glioma Database. F1=F1 score. NHNN=National Hospital for Neurology and Neurosurgery. UCSF-PDGM=University of California San Francisco Preoperative Diffuse Glioma Dataset. UPENN-GBM=University of Pennsylvania Glioblastoma Dataset.

Voxel-level out-of-sample segmentation performance for nnU-Net, expressed as mean ± SD Dice coefficient across different components, was as follows: normal brain (0·987 ± 0·024), non-enhancing abnormal tissue (0·821 ± 0·233), and enhancing tumour (0·574 ± 0·319, appendix pp 10, 13). When evaluating clinical performance thresholds among the 992 enhancing cases, we observed that 762 (77%) had a Dice score greater than or equal to 0·3 (acceptable detection), 670 (68%) had a Dice score greater than or equal to 0·5 (good detection), and 498 (50%) had a Dice score greater than or equal to 0·7 (excellent detection; appendix p 13).

Among the 992 enhancement-positive cases, voxel-level Dice varied widely across cohorts, being highest in research-grade glioma datasets (eg, UPENN-GBM) and lowest in the older real-world NHNN cohort and the paediatric BraTS-PEDs cohort. Performance stratified by pathology and country of origin (figure 5; appendix p 13) followed the same pattern, with the strongest performance being observed for meningioma, the weakest for paediatric tumours, and the lowest performance observed in the UK-based NHNN real-world dataset.

Enhancing tumour burden was accurately predicted by the model, with an R^2^ of 0·859 and a mean volumetric difference between prediction and the ground truth of −1·30 cm^3^ (figure 6). There was a clear relationship between the case-level detectability of an enhancing lesion and its corresponding Dice score. Lesion detectability was more variable for tiny lesions (approximately 0·01 cm^3^), with approximately 50% detection success, whereas lesions measuring 2 cm^3^ or larger were correctly detected in more than 90% of cases.

**Figure 6:**
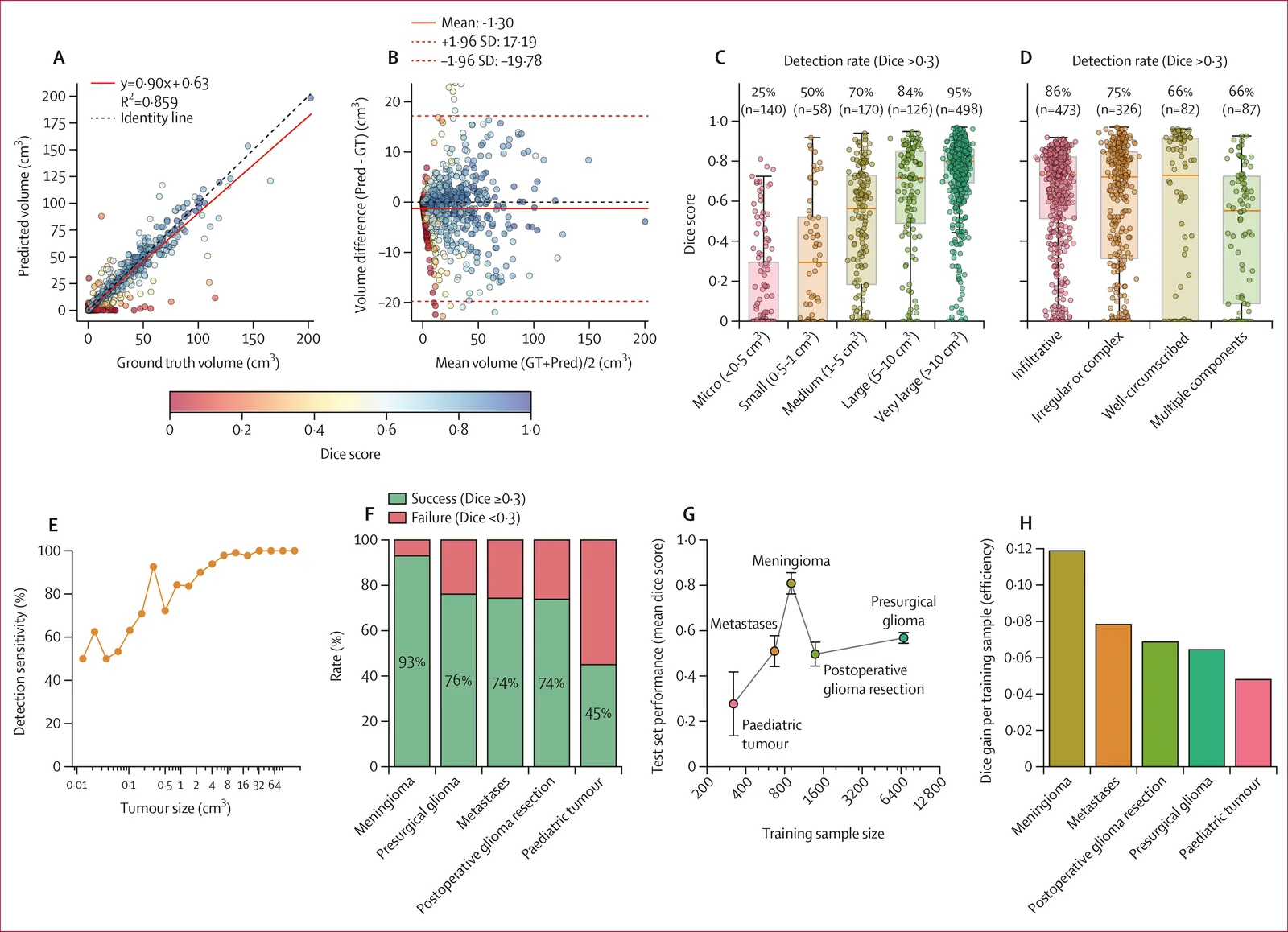
Relationship between model performance and lesion volume, morphology, and pathology (A) Volume correlation analysis showing strong agreement between predicted and ground-truth enhancement at the patient-level. (B) Bland–Altman plot assessing agreement between predicted and ground-truth enhancement volumes (patient-level), showing the mean difference (solid red line) and ±1·96 SD limits of agreement (red dashed lines). (C) Voxel-level segmentation performance (Dice coefficient) and detection success rate (annotated percentage; success defined as voxel-level Dice score ≥0·3) stratified by lesion-volume category. (D) Patient-level binary detection rate according to radiomics-derived morphological pattern. (E) Patient-level lesion detection sensitivity as a function of enhancement volume (proportion of cases where any model-predicted enhancing voxels overlapped with ground-truth enhancement). Panels (C) and (E) use different evaluation criteria (Dice ≥0·3 threshold *vs* binary overlap detection), which explains the apparent discrepancy in detection rates for small lesions. (F) Patient-level detection success (green) and failure (red) rates by pathology type. (G) Relationship between training sample size and test-set performance (Dice score) for each pathology type. (H) Performance efficiency, measured as Dice score gain per training sample, identifying pathologies with the highest learning efficiency. GT=ground truth. Pred=Predicted. SD=standard deviation.

As commonly reported in segmentation tasks, voxel-level performance varied with lesion volume (ANOVA p<0·0001; figure 6C), with Dice scores increasing monotonically from small to large lesions. Segmentation performance also varied according to radiomic characterisation of lesions (ANOVA p<0·0001; figure 6D). Lesion detectability was significantly associated with lesion size, expressed by the logistic regression model $y\;∼\;1\cdot 713\;\log_{10}\left(V\right)-4\cdot 786$, where V denotes lesion volume (p<0·0001; log-likelihood −361·464). The odds of lesion detection increased 5·546-fold (95% CI 4·389–7·009) for each order-of-magnitude (10×) increase in lesion volume (ie, each time the volume multiplied by ten). There was no statistically significant relationship between model segmentation performance and the number of available samples per pathology (data not shown).

Systematic examination of cases with poor segmentation performance (Dice <0·3) revealed several contributing factors. Most cases not reaching the acceptable detection threshold (Dice <0·3) were associated with small lesion volume (<0·5 cm^3^), followed by post-treatment cases with complex enhancement patterns (including surgical cavity enhancement), marked imaging artefact or suboptimal image quality in older acquisitions, and atypical enhancement patterns, including faint or incomplete rim enhancement, or absent enhancement in lesions ordinarily expected to enhance—a pattern particularly noted in paediatric cases. A quantitative breakdown of error categories is provided in the appendix (p 14). These observations suggest that model refinement should prioritise augmented training on challenging cases and potentially incorporate image quality assessment to identify unreliable predictions.

We also evaluated model probability maps (ie, before generating the binary segmentation map) to assess model uncertainty. Across the 1109 test-set cases, mean probability within the enhancing tumour prediction channel was 0·583 ± 0·301, with a mean entropy of 0·160 ± 0·089 (boundary fraction 0·075 ± 0·050); no cases showed high uncertainty (ie, with entropy >0·5; appendix p 7). Maximal predicted probabilities were high across pathologies. Levene's test for equality of variances showed a significant difference (p=0·0021), indicating that model uncertainty showed greater variance within pathology cohorts with smaller sample sizes. Variance in model uncertainty did not differ significantly across sex or age categories after correction for multiple comparisons.

## Discussion

We developed and evaluated a deep learning model for detecting and segmenting enhancing tumours from non-contrast MRI sequences alone. This study, to our knowledge, is the largest of its kind, using large-scale international data across ten different cohorts, five different disease categories, and four countries. Our findings provide strong support for the ability of complex models to identify and delineate enhancing intracranial disease from non-contrast MRI alone. Although contrast administration is widely considered necessary to detect enhancing disease, our finding that a model can predict enhancement with more than 83% accuracy from non-enhanced images alone suggests otherwise. Sufficiently capable artificial intelligence models might leverage information from pre-contrast imaging that human experts cannot perceive. Such models could provide a pathway towards reducing reliance on contrast agents.

We envision the predominant real-world utility of this model as a triage or decision-support adjunct rather than a complete replacement for contrast-enhanced imaging. Given the model's high sensitivity and the asymmetric cost of missing an enhancing lesion, the most defensible deployment strategy is as a gatekeeper for contrast administration. In workflows where only non-contrast sequences are protocolled, the model could flag patients in whom enhancement is likely so that a contrast-enhanced acquisition can be added, thereby avoiding a second hospital visit and minimising delays to patient care. Conversely, patients predicted not to have enhancing disease might, with appropriate caution, undergo imaging without gadolinium, particularly in settings where repeated imaging is required (eg, surveillance follow-up), where contraindications exist (renal impairment or allergy), or where minimising gadolinium exposure is prioritised (frequently repeated imaging). Any patient with an uncertain or positive prediction would proceed to standard contrast-enhanced imaging. Used in this manner, the tool could reduce gadolinium use while preserving the safeguard that any suspected enhancement triggers confirmatory contrast-enhanced imaging. The clinical value of this operating point is strongly dependent on prevalence. At the high enhancing tumour prevalence of our enriched test set (992 [89·4%] of 1109), the negative predictive value is low (0·510) but rises to approximately 0·95 at the lower, surveillance-realistic prevalences (≈30%) at which such a triage tool would most plausibly operate. The corresponding clinical utility estimates (contrast studies spared and enhancing tumours missed per 1000 scans) and full predictive values are provided in the appendix (pp 15–16). Predictive performance should therefore be interpreted against the prevalence expected in the intended deployment setting rather than that observed in the development cohort.

Our model’s performance, both as a patient-level lesion enhancement detector and a computer vision segmentation tool, is comparable to the performance reported in the broader literature. Previous internal research has shown a Dice segmentation performance of 0·79 for enhancing tumour delineation using T1w, T2w, and T2/FLAIR-weighted sequences alone in the more homogeneous BraTS 2021 glioma cohort.^4^ However, this was evaluated in a substantially smaller and more uniform dataset. Here, we report Dice segmentation performance for gliomas (cohort-dependent) of up to 0·824 on the UPENN-GBM dataset but observe more variable performance reflecting lesional and imaging heterogeneity and the quality of the available imaging data. Our deliberate inclusion of internal real-world clinical data (NHNN cohort), which dates back to 2006, shows substantial differences in imaging quality across MRI manufacturers, field strengths, and imaging parameters. Excluding this cohort raised rather than lowered overall performance (appendix p 17), indicating that its inclusion provides a more conservative estimate of overall performance. Other comparative studies include that by Kleesiek and colleagues, who developed a Bayesian U-Net using T1w, T2w, FLAIR, diffusion-weighted imaging (b0, b1000, and ADC), and susceptibility-weighted imaging (magnitude, phase, and minimum intensity projection) to produce virtual contrast maps in a cohort of 116 patients^22^ and reported strong discrimination for enhancing tumour detection in their validation set. By comparison, our model reached a balanced accuracy of 0·830 in an entirely held-out test set of 1109 studies using only three standard structural imaging sequences. The inclusion of diffusion-weighted and susceptibility-weighted imaging by Kleesiek and colleagues is particularly notable,^22^ as diffusion characteristics can assist radiologists in identifying tumour hypercellularity.^23^ Regarding methods of reducing gadolinium dose (but not eliminating it entirely, as attempted here), Ammari and colleagues reported 83% sensitivity for lesions larger than 10 mm using deep learning for virtual contrast prediction with a quarter of the standard gadolinium dose,^24^ an approach also explored by other groups.^5^^,^^25^ Murugesan and colleagues recently showed similar feasibility in a single-institution glioma cohort.^26^ Our study extends this approach to a substantially larger dataset (n=11 089) spanning multiple pathologies, age groups, and imaging protocols, thereby establishing broader generalisability. For direct comparison using consistent patient-level metrics, our model’s 77% success rate (proportion of cases reaching Dice ≥0·3) should be interpreted in the context of our deliberately heterogeneous cohort. Most previous studies used smaller, more homogeneous, single-institution datasets with larger lesions,^22^^,^^26^ in which Dice values can be inflated.^27^ Our cohort includes highly heterogeneous real-world data spanning ten datasets, five pathology types, four countries, and imaging from 2006–24, including older, lower-quality acquisitions from the NHNN cohort.

Although this diversity reduces the aggregate performance, it provides a more realistic assessment of generalisability. The variation in voxel-level Dice across cohorts reflects two distinct, at least partially separable factors. The first is distributional shift between sources—differences in scanner manufacturer, field strength, acquisition era, and annotation protocol, most pronounced in the older real-world NHNN data. The second is genuine differences in lesion characteristics, since cohorts differ systematically in lesion size, morphology, and pathology, and smaller or more diffuse lesions are intrinsically harder to segment. As in any machine-learning study, lower aggregate performance in a given cohort reflects both how far that cohort is from the training distribution and how challenging its lesions are to segment.

An important consideration is the nature of our comparison with human radiologists. The deep learning model was specifically trained on thousands of labelled examples to learn the mapping from non-contrast to contrast-enhancing appearances, whereas the radiologists were asked to perform a task outside their standard clinical training, without access to clinical history, previous imaging, or the referral indication. Radiologists were trained to interpret enhancement after gadolinium administration, not to predict it from intrinsic tissue characteristics alone. The radiologists’ performance on this task was therefore not a reflection of their diagnostic capability in routine practice, where enhancement is read directly from post-contrast images; it reflects only the difficulty of a task deliberately designed to lie outside their standard clinical training. What the result demonstrates, however, was that non-contrast sequences contain sufficient information to predict subsequent enhancement patterns in many cases, and that this ability was strongest in the artificial intelligence model, a finding with implications for reducing gadolinium dependence and for understanding the tissue properties that drive contrast uptake.

Equitable model performance across patient demographics and clinical subgroups is a prerequisite for clinical deployment of any such tool.^20^^,^^21^^,^^28^ Artificial intelligence is one of the fastest-growing fields in research, industry, and society, with numerous proposed applications in medicine.^29^^,^^30^ Yet, the equity of software systems is rarely quantified.^20^ For these reasons, we evaluated the performance equity of our model as granularly as possible across the largest and most diverse cohort available. Our findings suggest that the model performed similarly across patient age and sex, although performance was lower in paediatric populations. This weaker performance in paediatric cases is likely due, at least in part, to the substantially smaller number of paediatric cases in our cohort, with only 352 (3·17%) of 11 089 cases being paediatric and the remainder, adult. Although our cohort was large overall, paediatric patients were under-represented compared to adults because of limited sample availability. Another potential, and likely combinatorial, explanation is the inherent complexity of paediatric neuro-oncology, where enhancement patterns often differ substantially from those observed in adult lesions. Moreover, considerable heterogeneity exists within the paediatric population itself, especially across the spectrum of paediatric low-grade gliomas and glioneuronal tumours. A further strength of this study is the inclusion of highly heterogeneous data from a wide range of sources, which is likely to maximise its generalisability. This interpretation is supported by previous work highlighting the effects of distributional shift on model performance^31^^,^^32^ and by studies highlighting the potential of approaches such as federated learning or expanded data sharing to mitigate these effects.^32^

An alternative approach to predicting enhancement could use quantitative parametric maps (eg, T1/T2 relaxometry, cerebral blood volume perfusion imaging) instead of, or in addition to, qualitative weighted images. Such maps offer potential advantages: they are more standardised across scanners and directly measure tissue properties associated with blood–brain barrier disruption, the principal mechanism underlying contrast enhancement. However, quantitative parametric maps are often not routinely acquired in clinical practice, require longer acquisition times and additional post-processing, and are less available in older or resource-limited datasets. Notably, Kleesiek and colleagues^22^ included diffusion-weighted and susceptibility-weighted imaging in their model and showed strong discrimination in a much smaller cohort, whereas our model reached a balanced accuracy of 0·830 using only standard clinical sequences (T1w, T2w, T2/FLAIR), suggesting that substantial enhancement-predictive information is already present in conventional structural images. We encourage future work to investigate whether supplementing standard sequences with quantitative maps further improves performance, particularly for small or subtle lesions.

We deliberately framed this problem as segmentation rather than image-to-image synthesis or whole-image classification. A classifier returns only a study-level label and cannot localise disease, whereas synthesis of a full virtual post-contrast image is subject to a far more demanding standard, requiring faithful reproduction of every voxel of intensity and morphology—and current evidence indicates that generative synthesis has not yet reached the fidelity required for clinical use. Morphology-preserving generative approaches^33^ remain research tools rather than deployable diagnostics, and large foundation efforts such as NV-Generate-MR-Brain^34^ have shown plausible synthesis that has not yet been clinically validated. Segmentation occupies a useful middle ground: it simultaneously classifies (whether enhancing disease is present) and delineates (where it is), yielding an interpretable, spatially grounded output that is directly auditable against ground-truth enhancement without staking diagnostic safety on the fidelity of a synthesised image.

Our study has some limitations. First, the cohort disproportionately included adult rather than paediatric patients and larger rather than smaller lesions. Our research prioritised maximal inclusivity in all modelling tasks. To that end, the inclusion of any paediatric or small-lesion samples (even when markedly under-represented relative to adult cohorts) was considered preferable to excluding this population from analysis. Future work should include larger cohorts with more diverse caseloads to improve model performance in these subpopulations, whether through de novo model training or fine-tuning using weights derived from adult data. A strength of our study is that we tested these issues empirically as a key component of the equity assessment. We also included all available data, encompassing global data from several countries and diverse clinicodemographic populations, thereby providing greater generalisability than is typically reported in comparable studies. Second, although our model showed strong sensitivity (0·915) for detecting an enhancing lesion on unenhanced images, its specificity was lower (0·744), resulting in more false positives than false negatives. Future research could improve specificity by incorporating additional data from further cohorts, particularly non-enhancing tumours, which were relatively under-represented in our dataset. Importantly, one core purpose of such a model would be to classify imaging sessions in which lesions are highly likely to enhance, allowing post-gadolinium imaging to be omitted from the initial scan, follow-up imaging, or both. In this context, prioritising sensitivity over specificity is arguably preferable. A further limitation concerns the interpretation of the model's predicted probabilities. We report these as a descriptive characterisation of predictive entropy rather than as calibrated probabilities. Formal calibration (eg, temperature scaling assessed using reliability diagrams) was not undertaken in this study. Post-hoc calibration would be essential before predicted probabilities could be used to guide uncertainty-based deployment decisions and should be prioritised in future work. Third, performance was weakest for small lesions, which accounted for the largest population of undetected lesions. This finding partly reflects internal class imbalance, as small enhancing targets were under-represented relative to larger lesions but is also likely attributable to the known behaviour of Dice loss,^27^ whereby overlap-based losses disproportionately penalise small-structure errors and bias models away from tiny targets. Future work should consider size-aware sampling, complementary detection-oriented loss functions, and lesion-size-stratified reporting.

In conclusion, we demonstrate that deep learning models can identify contrast-enhancing intracranial disease in neuro-oncology when exposed to the non-contrast MRI sequences alone. That a deep learning model can identify areas of pathological enhancement from non-enhanced images supports the potential of artificial intelligence approaches to generate synthetically enhanced or low-dose enhanced post-contrast comparators.^5^, ^6^, ^7^ In their current form, such approaches could be particularly valuable as decision-support or triage tools that minimise, rather than completely replace, the use of contrast-enhanced imaging. However, we hope that future increases in data availability, algorithmic complexity, and high-performance computing will create further opportunities for innovation in this area. These findings might also advance understanding of the radiological basis of lesional enhancement, contribute to radiological pedagogy,^35^ and highlight the potential of deep learning to elucidate imaging features that could ultimately reduce or even eliminate the need for contrast agents.

## Data sharing

All imaging datasets used in this study, with the sole exception of the NHNN legacy cohort, are openly available under the terms of their original licences, and can be obtained from their respective repositories (BraTS-GLI, BraTS-MEN, BraTS-METS, BraTS-PEDs, BraTS-SSA, BraTS 2021, EGD, UCSF-PDGM, and UPENN-GBM).^8^, ^9^, ^10^, ^11^, ^12^, ^13^, ^14^, ^15^, ^16^ The NHNN cohort cannot be shared because the governing research ethics approval does not permit onward dissemination of these clinical images. Model weights, the inference pipeline, and the code used for training, evaluation, and analysis are openly available in the GitHub repository.

## Declaration of interests

GP is affiliated with NVIDIA but contributed to this work as an academic collaborator and former core member of the research team, and not in any capacity related to NVIDIA. NVIDIA had no involvement in the research, including study design, data collection, analysis, interpretation, or the decision to publish, and provided no hardware or other in-kind contributions. PN is affiliated with Hologen, a healthcare artificial intelligence company, which had no involvement in this research. All other authors declare no competing interests.
