# Supplementary material for: Predicting brain tumour enhancement from non-contrast MRI with artificial intelligence: a multicohort, retrospective, diagnostic accuracy study
Source: Lancet Med Imaging Theranostics. 2026 Sep;1(2):None. doi: 10.1016/j.lanmit.2026.100012 (PMC13637711; doi:10.1016/j.lanmit.2026.100012)
Supplement: Supplementary file 1 [file mmc1.pdf]

THE LANCET
Medical Imaging & Theranostics

Supplementary appendix

This appendix formed part of the original submission and has been peer reviewed.
We post it as supplied by the authors.

Supplement to: Ruffle JK, Mohinta S, Pombo G, et al. Predicting brain tumour enhancement from non-contrast MRI with artificial intelligence: a multicohort, retrospective, diagnostic accuracy study. *Lancet Medical Imaging & Theranostics* 2026. //doi.org/10.1016/j.lanmit.2026.100012

Predicting brain tumour enhancement from non-contrast MR imaging with artificial intelligence: a multi-cohort retrospective diagnostic accuracy study

Appendix

Contents

Methods 1
Results 6
Supplementary Figure 1. Data partitioning characteristics 6
Supplementary Figure 2. Model discriminative performance analysis 7
Supplementary Table 1. Cohort demographics and baseline characteristics 8
Supplementary Table 2. Patient-level enhancement detection performance 9
Supplementary Table 3. Voxel-wise segmentation performance by architecture 10
Supplementary Table 4. Patient-level confusion matrices 11
Supplementary Table 5. Balanced accuracy by architecture and subgroup 12
Supplementary Table 6. Voxel-level segmentation performance by cohort 13
Supplementary Table 7. Failure category breakdown by cohort 14
Supplementary Table 8. Clinical utility across prevalences 15
Supplementary Table 9. Negative and positive predictive value by prevalence 16
Supplementary Table 10. Patient-level performance with and without NHNN 17
References 18

Methods

Data

Local data

We included 3905 imaging sessions from 1364 unique patients who were imaged and treated for a glioma at our centre, the National Hospital for Neurology and Neurosurgery (NHNN), between 2006 and 2024, drawn from prospective histopathology record-keeping within the Institute's Department of Neuropathology. Having delineated a viable neuropathology cohort[1], we retrieved all magnetic resonance imaging (MRI) scans from the Picture Archiving and Communications System (PACS). We also recorded diagnostic and demographic data in these individuals.

External data

We acquired multiple open-source datasets to boost sample sizes and maximise the generalizability of results in neuro-oncology internationally. The datasets and sample sizes used were as follows: 1) The Brain Tumor Segmentation Challenge (BraTS) (n=1470)[2]; 2) the Erasmus Glioma Database (EGD) (n=774)[3]; 3) The University of Pennsylvania Glioblastoma (UPenn-GBM) cohort (n=665)[4]; 4) The University of California San Francisco Preoperative Diffuse Glioma (UCSF-PDGM) cohort (n=501)[5], 5) The Brain Tumor Segmentation – Post-treatment Challenge (n=1538)[6], 6) The Brain Tumor Segmentation – Metastases Challenge (n=739)[7], 7) The Brain Tumor Segmentation – Paediatric Challenge (n=352)[8], 8) The Brain Tumor Segmentation – Meningioma Challenge (n=1000)[9], and 9) The Brain Tumor Segmentation – Glioma in Sub-Saharan Africa Challenge (n=145)[10]. In instances of crossover between datasets (such as the inclusion of UCSF-PDGM[5]and UPenn-GBM[4], which both feature, in part, in BraTS 2021[2]), we contacted the principal investigators of each study to clarify those specific cases and ensure that no participants were erroneously duplicated in our set. As a secondary quality control measure, any lesion whose appearance was highly correlated (voxel-wise $r > 0.95$) was flagged for cross-checking to ensure no duplicate participants. Paired demographics were variably available for these external repositories, the completeness of which was beyond our control.

Image pre-processing

We acquired all available MRI sequences for all available imaging sessions from our local neuro-oncology cohort, with data ranging from 2006 to 2024. We deployed a locally developed MRI sequence classifier and artefact detector for initial image quality control and to curate imaging sequences[11]. We clamped MRI signal intensities at the 1st - 99th percentile to attenuate any spurious signal artefacts. All sequences were super-resolved to 1x1x1mm isotropic resolution using a locally developed generative super-resolution model[12]. Images were brain extracted with HD-BET[13], from which the brain extraction masks were also curated. The three non-contrast input sequences (T1-weighted, T2-weighted, and FLAIR-weighted images) were concatenated as separate channels along a fourth tensor dimension, providing each model with a four-dimensional multi-channel volumetric input.

We manually reviewed all raw data at all stages of the data pre-processing. Intensity- and scanner-harmonisation tools (for example, ComBat-style site/scanner normalisation) were considered but deliberately not applied. Because the central aim was generalisation across heterogeneous sites, scanners, and acquisition protocols, we elected to retain the inter-site

and inter-scanner signal present in the multi-cohort data rather than regress it out, so that the model was trained and evaluated on the full distributional variability it is intended to encounter in deployment. Moreover, though harmonisation may well assist this task, harmonisation models can also introduce unpredictability in the context of pathology.

Ground truth derivation

Ground-truth enhancing-tumour masks were always generated from complete datasets — non-contrast T1-, T2-, and T2/FLAIR-weighted together with post-contrast T1-weighted imaging — which were available for all patients. For the external open-source datasets, the challenge-standard expert annotations supplied with each dataset were used directly; these were produced independently of our group. For the internal NHNN cohort, ground-truth labels were generated in-house with TumourSeg[14], our previously published, openly available tumour-segmentation model, whose architecture, weights, and training data are entirely separate from the three enhancement-prediction architectures evaluated here; every inferred NHNN label was then reviewed, and fine-tuned where required, by an expert neuroradiologist with seven years of neuro-oncology experience (JKR).

Whilst the NHNN labels were thus produced by a related in-house model and adjudicated by a single reviewer — which could in principle bias the NHNN voxel-level performance estimate — the patient-level presence or absence of pathological contrast enhancement was deemed plausibly objective and unbiased, since detecting it from post-contrast imaging is a standard radiological reporting skill. Critically, the 1,109 test cases were strictly held out from all training, including the training of the label-generation model, so that no test case contributed to either enhancement-prediction training or ground-truth-label generation.

Background (non-brain) labels were derived from brain extraction with HD-BET[13]. All sequences within an imaging session were acquired during the same scanning session, pre-processed using gold-standard approaches, and co-registered to MNI space (see *Image pre-processing*). Cases with substantial inter-sequence motion or registration failure on visual quality control were excluded. For post-treatment cases, surgically related enhancement was distinguished from tumour enhancement by expert neuroradiological consensus within the source datasets.

Nonlinear anatomical registration with enantiomorphic normalisation

Having segmented lesions in the patient's native space, structural and lesion segmentation masks were nonlinearly registered to 1x1x1mm isotropic resolution MNI space with Statistical Parametric Mapping (SPM, v12) and enantiomorphic correction[15,16]. The advantage of enantiomorphic correction is that the risks of registration errors secondary to a lesion are minimised by leveraging a given patient's normal structural neuroanatomy on the unaffected contralesional hemisphere[15].

Model development

Model architectures trained included nnU-Net (v2)[17], SegResNet[18], and SwinUNETR[19,20]. For nnU-Net, we trained the default 1000-epoch model and a 4000-epoch model with the large residual connection encoder modification, which represents a reasonable balance between computational cost and performance gain[21]. For SegResNet[18], architectural parameters

included 16 output channels for the initial convolution layer, a dropout probability of 0.1, and ReLU activation. For SwinUNETR[19,20], architectural parameters included a feature size of 48, a dropout and attention dropout rate of 0.1. We used a learning rate of 0.001, the Adam optimiser, a cosine annealing learning scheduler, and Dice loss. For both SegResNet and SwinUNETR, we enabled a variety of augmentations, including foreground cropping and signal normalisation, random axis flipping, coarse dropout, intensity shift and scaling, bias field, histogram shifts, Gaussian smoothing, low resolution simulation, affine and 3D elastic deformations[22]. Data augmentations were enabled within nnU-Net per its creators' standard settings[17]. SegResNet and SwinUNETR models were trained for up to 4000 epochs, but with an early stopping patience of 100 epochs. Hyperparameters were tuned only within the training set, using internal validation of this set. For SegResNet and SwinUNETR, the dropout rate (0.1) and learning rate (0.001) reported above were selected on the basis of enhancing-tumour Dice achieved on the internal held-out validation split, rather than on any test-set performance. All three architectures were trained from random initialisation, with no pretrained weights used for any model. This eliminates the possibility of pretraining-derived data leakage, since no weights were derived from data overlapping the held-out test set. Model training was conducted on a Linux GPU-enabled workstation (NVIDIA RTX 6000 Ada Generation). Training times for a dataset of this size were substantial, at approximately 2000 hours (~83 days) per model. Total compute and training-hour budgets were matched across the three architectures. Within that fixed budget, nnU-Net's optimised data-loading and I/O permitted its default internal 5-fold cross-validation, whereas SegResNet and SwinUNETR were trained on a single held-out validation split due to compute demand constraints. We acknowledge that this does not constitute strict tuning-budget parity, since the architectures differed in how the matched budget was apportioned between cross-validation folds and training epochs.

Evaluation

Performance metrics

We performed a randomised train-test split at the patient level, with 90% of patients allocated to training and 10% to model testing, ensuring no leakage between sets. The test set was locked until all model development was complete. For the NHNN cohort, where individual patients contributed multiple imaging sessions (up to 3-4 examinations per patient), the split was performed strictly at the patient level, ensuring that all examinations from a given patient were assigned to the same partition and maintaining complete patient-level independence between training and test sets. The unit of analysis was the imaging session rather than the patient; where a patient contributed more than one study (for example, separate pre- and post-treatment acquisitions), these represent distinct imaging presentations and were treated as independent observations. This partition was fixed for all model training runs to ensure maximal comparability. A leave-one-cohort-out (LOCO) training and evaluation scheme was considered but not adopted on the grounds of model training/inference equity. Excluding a cohort such as the Sub-Saharan Africa or paediatric datasets from training would have primed the model to underperform in precisely the populations in which equitable performance matters most, whereas excluding such a cohort from the test set would have removed the only empirical evidence of performance in those groups. We therefore retained all cohorts in both training and testing and instead offer cohort-stratified performance as a partial proxy for the cross-cohort generalisation that LOCO is intended to probe. This maximally inclusive, general-purpose design mirrors the trajectory of the BraTS challenge, which has moved from separate disease-specific tasks towards a unified 'Generalizability Across Tumours' objective;

conversely, training separate models per cohort would require at least six distinct networks (pre-treatment (and pre-operative) glioma, post-treatment glioma, Sub-Saharan low-field imaging, meningioma, metastases, and paediatric) and would forfeit the cross-cohort robustness that motivated pooling.

Metrics were formally defined as follows: Dice coefficient = 2|P ∩ G| / (|P| + |G|); balanced accuracy = (sensitivity + specificity) / 2; sensitivity (recall) = TP / (TP + FN); specificity = TN / (TN + FP); precision = TP / (TP + FP); F1 = 2 × (precision × recall) / (precision + recall). For each metric, we specify whether it is computed at the patient level (binary classification of enhancement presence) or voxel level (spatial segmentation accuracy). Detection metrics (balanced accuracy, sensitivity, specificity, precision, and F1 score) were computed only for the enhancing class, as this was the primary clinical target. Balanced accuracy and macro-averaged metrics were used throughout because they are the standard, bias-resistant choice for evaluating class-imbalanced detection tasks[23,24]. The Dice coefficient was reported for all three foreground tissue classes (normal brain parenchyma, non-enhancing abnormal tissue, and enhancing tumour).

Equity assessment

Equity of model performance was assessed by disaggregating diagnostic performance across all available demographic and clinical subgroups — contributing dataset, site, country of acquisition, tumour diagnosis, patient age and sex — following the Fairboard framework[25]. Within each subgroup, we report the same patient-level detection metrics used throughout (balanced accuracy, sensitivity, specificity, precision, recall and F1, together with the area under the receiver-operating-characteristic curve), so that performance can be compared directly across groups. Subgroups containing only enhancement-positive patients have undefined specificity, in which case balanced accuracy reduces to sensitivity. No subgroup-specific probability recalibration was performed. We also quantified the model detection rate in relation to radiomic features of enhancement, determining the relationship between segmentation performance and whether it was infiltrative, irregular/complex, well-circumscribed, solitary or multiple. The radiomic categories were defined based on quantitative shape features extracted from 3D binary masks of enhancing brain lesions. The following features were calculated: 1) Number of connected components from 3D connected component analysis; 2) Sphericity, calculated as $\frac{\pi^{\frac{1}{3}}(6V)^{\frac{2}{3}}}{A}$, where V denotes volume and A is surface area; 3) Solidity: calculated as the ratio of actual lesion volume to convex hull volume; 4) Compactness, calculated as $\frac{A^3}{(36\pi V^2)}$, where A is surface area and V is volume; 5) Surface-to-Volume Ratio, the surface area divided by volume; and 6) Elongation, ratio of largest to smallest eigenvalues from principal component analysis of lesion voxel coordinates. Based on these quantitative features, lesions were classified into the following radiomic categories: 1) Multiple – imaging sessions with ≥3 connected components, or two components where the largest component represents <80% of total enhancement volume; 2) Well-circumscribed single - a single dominant lesion with sphericity > 0.7 and solidity > 0.9; 3) Infiltrative single - a single dominant lesion with sphericity < 0.5 or solidity < 0.7; 4) Irregular/complex single – a single dominant lesion with intermediate features of sphericity 0.5 - 0.7 and solidity 0.7 - 0.9.

Software use

The following Python software and models were used for model development and downstream analyses: Fairboard[25], Matplotlib[26], MONAI[27], Nibabel[28], Nilearn[29], NumPy[30], pandas[31], PyTorch[32], seaborn[33], scikit-learn[34], and TumourSeg[14].

Results

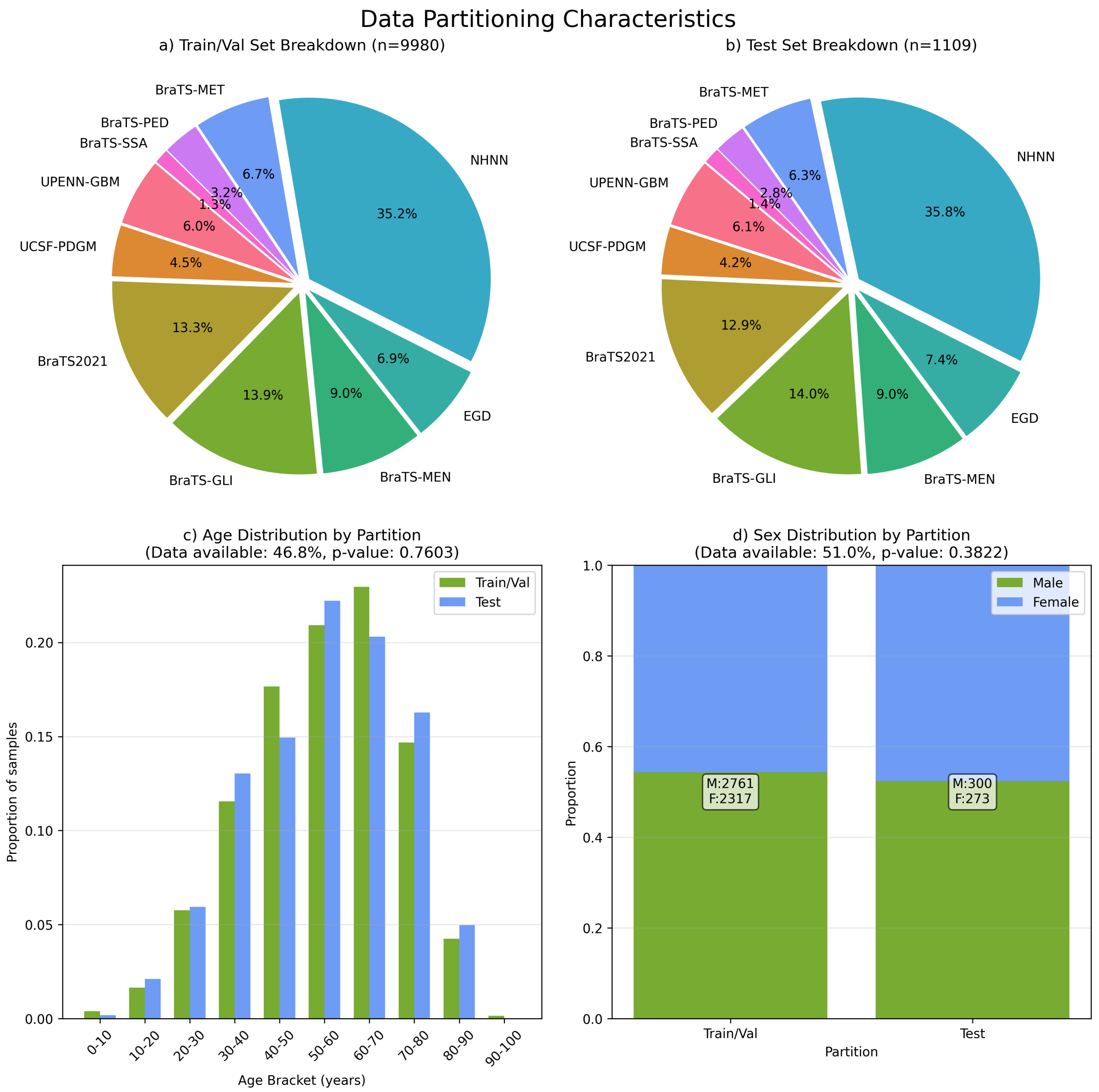

Supplementary Figure 1. Data partitioning characteristics. a) Training and validation set composition (n=9,980) showing proportional representation across all cohorts. b) Test set composition (n=1,109), maintaining equivalent proportions to training data, ensuring unbiased evaluation. c) Age distribution comparison between training/validation and test set partitions. d) Sex distribution comparison between training/validation and test set partitions.

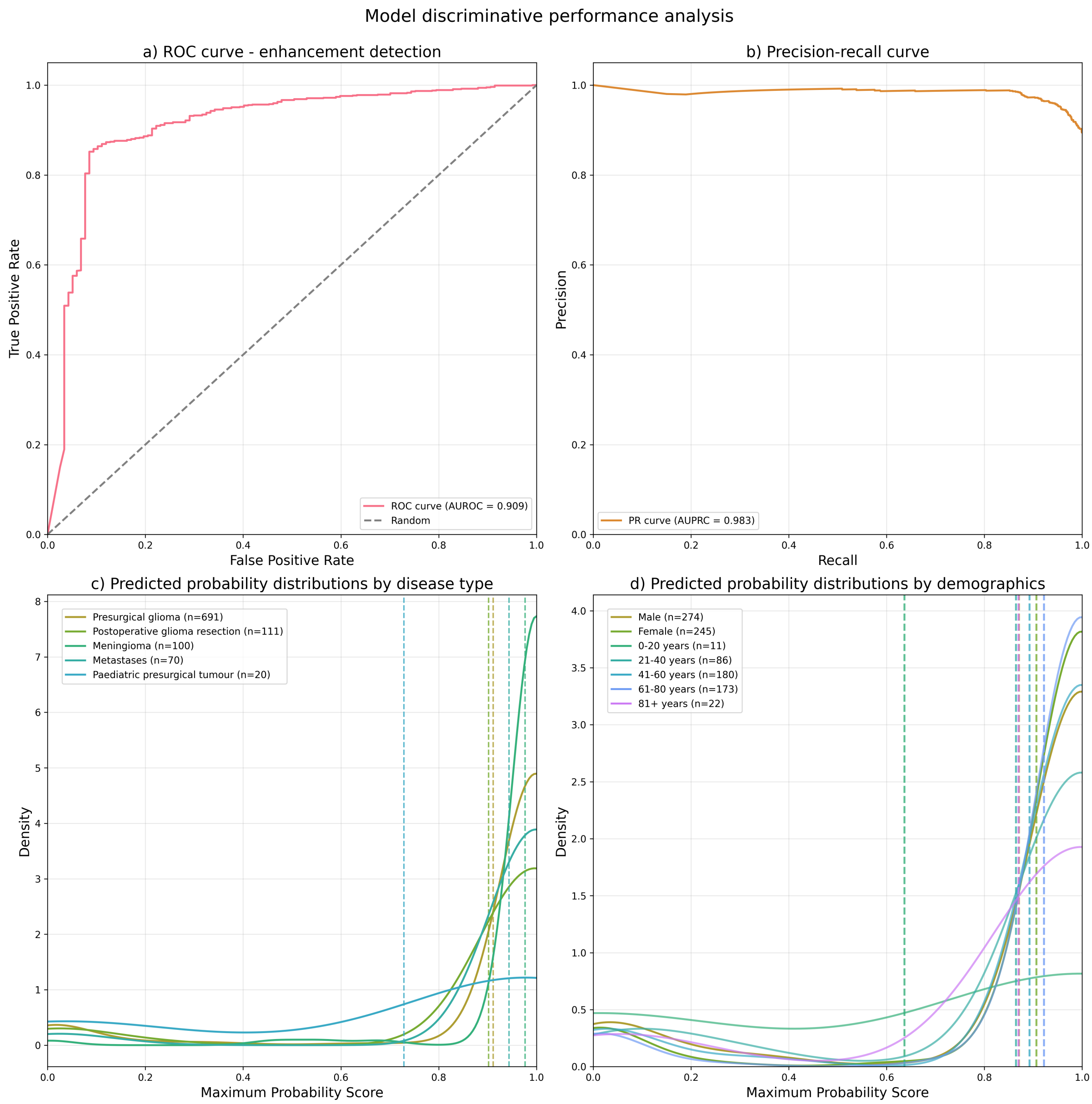

Supplementary Figure 2. Model discriminative performance analysis. a) Receiver Operating Characteristic (ROC) and b) Precision-Recall curve for enhancement detection. c-d) Predicted probability distributions stratified by c) pathology type and d) demographics.

Patient-level detection differences were confirmed by paired case-level bootstrap: nnU-Net exceeded SegResNet by a balanced-accuracy difference of +0.081 (95% CI 0.026–0.137, p=0.0034) and SwinUNETR by +0.119 (0.063–0.174, p<0.0001) (Supplementary Table 2). This advantage was driven by sensitivity (nnU-Net vs SegResNet Δsensitivity +0.154, p<0.0001; vs SwinUNETR +0.169, p<0.0001) and F1 (+0.092 and +0.105 respectively, both p<0.0001), whereas specificity differences were not significant (vs SegResNet +0.009, p=0.94; vs SwinUNETR +0.068, p=0.24). The patient confusion matrices for enhancement detection between the model and the radiologist are also provided below in Supplementary Table 4.

Cohort	Country	Pathology	Imaging sessions (train/test)	Unique patients	Age available, n (%)	Age, mean (SD), y	Sex (M/F/Unknown)
UPENN-GBM	USA	Glioma	665 (597/68)	665	665 (100%)	62.4 (12.4)	402/263/0
UCSF-PDGM	USA	Glioma	501 (454/47)	501	501 (100%)	56.9 (15.0)	299/202/0
BraTS 2021	USA	Glioma	1470 (1327/143)	1470	0	N/A	0/0/1470
BraTS-GLI	USA	Glioma (post-treatment)	1538 (1383/155)	1538	0	N/A	0/0/1538
BraTS-MEN	USA	Meningioma	1000 (900/100)	1000	0	N/A	0/0/1000
EGD	Netherlands	Glioma	774 (692/82)	774	725 (93.7%)	56.7 (14.6)	492/281/1
NHNN	UK	Glioma	3905 (3508/397)	1364	3296 (84.4%)	52.8 (17.2)	1868/1844/193
BraTS-METS	USA	Metastases	739 (669/70)	739	0	N/A	0/0/739
BraTS-PEDs	USA	Paediatric	352 (321/31)	352	0	N/A	0/0/352
BraTS-SSA	Nigeria	Glioma	145 (129/16)	145	0	N/A	0/0/145
Overall	—	Mixed	11,089 (9,980/1,109)	8,548	5,187 (46.8%)	55.0 (16.4)	3,061/2,590/5,438

Supplementary Table 1. Cohort demographics and baseline characteristics. Age is in years, mean (SD), among sessions with recoverable age ('Age available'); the public BraTS challenge cohorts are age- and sex-stripped at source. Sex is shown as male/female/unknown.

Metric	nnU-Net	SegResNet	SwinUNETR	Expert radiologists (n=11)	Expert radiologists (majority vote)
Balanced accuracy	**0.830 ± 0.021 (0.791–0.872)**	0.748 ± 0.021 (0.705–0.786)	0.711 ± 0.023 (0.666–0.756)	0.698 ± 0.072 (0.654–0.738)	0.717 (0.670–0.763)
Sensitivity (recall)	**0.915 ± 0.009 (0.899–0.931)**	0.762 ± 0.013 (0.736–0.787)	0.746 ± 0.013 (0.720–0.772)	0.749 ± 0.076 (0.704–0.787)	0.776 (0.739–0.814)
Specificity	**0.744 ± 0.040 (0.667–0.825)**	0.735 ± 0.040 (0.654–0.810)	0.676 ± 0.043 (0.591–0.758)	0.647 ± 0.151 (0.562–0.736)	0.658 (0.570–0.737)
Precision	**0.968 ± 0.006 (0.957–0.979)**	0.961 ± 0.007 (0.946–0.973)	0.951 ± 0.008 (0.936–0.967)	0.680 ± 0.091 (0.631–0.733)	0.897 (0.865–0.925)
F1 score	**0.941 ± 0.005 (0.931–0.952)**	0.849 ± 0.009 (0.831–0.867)	0.836 ± 0.009 (0.819–0.854)	0.713 ± 0.056 (0.679–0.743)	0.832 (0.805–0.859)

Supplementary Table 2. Patient-level enhancement detection performance on the held-out test set of 1,109 studies. Model values are the bootstrap mean ± SD with the percentile bootstrap 95% CI in parentheses (1,000 resamples, seed 42), computed on the full 1,109-study test set at the deployed decision rules (nnU-Net, >0 predicted enhancing voxels; SegResNet and SwinUNETR, Youden-optimal volume thresholds). Expert radiologists (n=11) are the aggregate of 1,100 blind reads (11 readers × 100 cases each), pooled across reads, with SDs per-rater and CI by cluster bootstrap over readers. Expert radiologists (majority vote) are the per-case majority vote across blind reads on the 564-case reader-matched subset.

Metric	nnU-Net	SegResNet	SwinUNETR
Dice coefficient ↑	**0.574 ± 0.319**	0.523 ± 0.288	0.421 ± 0.277
Jaccard index (IoU) ↑	**0.467 ± 0.290**	0.402 ± 0.251	0.306 ± 0.226
Sensitivity (recall) ↑	0.581 ± 0.337	0.627 ± 0.286	**0.635 ± 0.297**
Precision ↑	**0.709 ± 0.259**	0.516 ± 0.308	0.389 ± 0.291
Hausdorff distance, mm ↓	**11.00 [6.56–22.60]**	16.43 [9.62–41.45]	30.71 [13.93–89.60]
95% Hausdorff distance, mm ↓	**4.01 [2.24–9.91]**	6.16 [3.61–16.85]	11.57 [5.74–31.88]
Average symmetric surface distance, mm ↓	**1.25 [0.71–2.83]**	2.03 [1.22–5.23]	3.97 [1.97–10.21]
Mean surface distance, mm ↓	**1.18 [0.69–2.47]**	1.95 [1.19–4.53]	3.66 [1.92–8.10]

Supplementary Table 3. Voxel-wise segmentation performance for the predicted enhancing-tumour class, by architecture. Voxel-wise segmentation performance for the predicted enhancing-tumour class on the 1,109-study held-out test set, comparing the three candidate architectures. Overlap and voxel-classification metrics (Dice, Jaccard/IoU, sensitivity, precision) are mean ± SD over all test samples with ground-truth enhancement. Distance metrics (Hausdorff, 95% Hausdorff, average symmetric surface distance, mean surface distance) are medians [IQRs], in mm, across studies in which all three models produced a non-empty enhancement prediction. Arrows indicate the favourable direction and bold marks the best value per row. Overlap (Dice/IoU) and voxel counts were obtained using the nnU-Net evaluation routine; surface-distance metrics were obtained using SimpleITK (exact Maurer distance transform). nnU-Net was superior to both SegResNet and SwinUNETR on Dice, IoU, precision and all four distance metrics (paired Wilcoxon signed-rank, all $p<0.0001$); voxel sensitivity did not differ significantly between the networks (p=0.46 and p=0.13); the comparators nonetheless tend to over-segment, evidenced by their substantially lower precision and larger surface distances.

A. nnU-Net model

GT ↓ / Prediction →	Model: enhancement	Model: no enhancement
GT: enhancement (n=447)	412	35
GT: no enhancement (n=117)	30	87

B. Expert radiologists (per-case majority vote)

GT ↓ / Prediction →	Radiologists: enhancement	Radiologists: no enhancement
GT: enhancement (n=447)	347	100
GT: no enhancement (n=117)	40	77

Supplementary Table 4. Patient-level confusion matrices for binary enhancement detection. Test computed on the 564 unique test cases reviewed by the expert-radiologist panel (447 GT-positive, 117 GT-negative). (A) nnU-Net model. (B) Radiologist majority vote (blind reads, no AI assistance; 359 single-rater, 205 multi-rater cases). **N.B.** (i) Because each radiologist independently sampled 50 enhancement-positive and 50 enhancement-negative cases, the 564 unique cases reviewed contain a higher proportion of ground-truth-negative cases than the full 1,109-study test set (20.7% vs 10.6% GT-negative). This enrichment accounts for the small difference between the model's balanced accuracy here (0.833) and in Supplementary Table 2 (0.830), where the full test-set prevalence applies. (ii) Of the 564 cases, 359 were reviewed by a single radiologist and 205 by two or more. Among the 205 multi-reviewed cases, raters were unanimous in 114 (55.6%) and disagreed in 91 (44.4%). Pairwise inter-rater agreement was fair (Cohen's κ = 0.36; observed agreement 68.3%), consistent with the difficulty of predicting enhancement from non-contrast imaging alone. (iii) 23 cases had an even number of raters split exactly 50/50, which in the main analysis were resolved as 'positive' (enhancement predicted) to emulate maximal clinical sensitivity.

Row	n	BA (95% CI)
**Model architecture**		
nnU-Net	1,109	0.830 (0.791–0.872)
SegResNet	1,109	0.748 (0.705–0.786)
SwinUNETR	1,109	0.711 (0.666–0.756)
**Subgroup (*nnU-Net only*)**		
Paediatric (BraTS-PEDs)	31	0.739 (0.555–0.900)
NHNN	397	0.790 (0.717–0.856)
Under 30 (exact age)	85	0.765 (0.577–0.906)
30 and over (exact age)	480	0.833 (0.764–0.893)
Male	303	0.816 (0.721–0.894)
Female	270	0.830 (0.738–0.909)

Supplementary Table 5. Balanced accuracy with 95% CI by architecture and subgroup. Patient-level balanced accuracy with percentile bootstrap 95% CIs (seed 42, 1,000 resamples). Architecture rows compare the three candidate networks, whereas the subgroup rows are computed for the deployed nnU-Net model.

Cohort	N	Dice (mean ± SD)	Mean Dice (95% CI, bootstrap)	Successful detection rate (Dice ≥ 0.3)	Successful detection rate (Dice ≥ 0.5)	Successful detection rate (Dice ≥ 0.7)
UPENN-GBM	68	0.824 ± 0.085	0.804–0.843	100.0%	98.5%	94.1%
BraTS-MEN	100	0.809 ± 0.239	0.756–0.852	93.0%	91.0%	85.0%
BraTS 2021	139	0.766 ± 0.185	0.735–0.795	96.4%	91.4%	83.5%
BraTS-SSA	16	0.717 ± 0.151	0.642–0.781	100.0%	87.5%	81.2%
UCSF-PDGM	43	0.704 ± 0.304	0.609–0.790	83.7%	83.7%	76.7%
EGD	75	0.533 ± 0.334	0.452–0.605	73.3%	68.0%	50.7%
BraTS-METS	70	0.510 ± 0.291	0.441–0.578	74.3%	64.3%	30.0%
BraTS-GLI	111	0.496 ± 0.283	0.444–0.553	73.9%	61.3%	29.7%
NHNN	350	0.423 ± 0.313	0.391–0.456	62.0%	46.9%	26.3%
BraTS-PEDs	20	0.277 ± 0.321	0.138–0.418	45.0%	35.0%	15.0%
**Total (weighted)**	**992**	**0.574 ± 0.319**	0.554–0.594	**76.8%**	**67.5%**	**50.2%**

Supplementary Table 6. Voxel-level segmentation performance (enhancing-tumour Dice) on the held-out test set. Table stratified by source cohort (n = 992 ground-truth-positive cases; 117 GT-negative cases excluded as Dice is undefined without ground-truth enhancement). Bootstrap 95% CIs on the mean.

Cohort (test-set N)	False positive	Small lesion (<0.5 cm³)	Paediatric	Post-treatment	Other/atypical	Total	Rate (%)
**BraTS-PEDs (n=31)**	3	3	8	0	0	14	45.2
**NHNN (n=397)**	14	62	0	0	71	147	37.0
**BraTS-GLI (n=155)**	11	12	0	17	0	40	25.8
**BraTS-METS (n=70)**	0	9	0	0	9	18	25.7
**EGD (n=82)**	0	11	0	0	9	20	24.4
**UCSF-PDGM (n=47)**	1	5	0	0	2	8	17.0
**BraTS-MEN (n=100)**	0	3	0	0	4	7	7.0
**BraTS 2021 (n=143)**	1	0	0	0	5	6	4.2
**UPENN-GBM (n=68)**	0	0	0	0	0	0	0.0
**BraTS-SSA (n=16)**	0	0	0	0	0	0	0.0
**Total (N=1109)**	30	105	8	17	100	260	23.4

Supplementary Table 7. Failure category breakdown by cohort on the held-out test set. Counts of test-set cases from the 1,109-study held-out set whose enhancement detection and voxel-level Dice for the enhancing-tumour class fell below 0.3 (n = 260 of 1,109 failing cases; cohort denominators are shown in the column headers).

Prevalence	Sensitivity	Specificity	PPV	NPV	Contrast administrations avoided per 1,000	Enhancing tumours missed per 1,000
89.4% (test)	0.915	0.744	0.968	0.510	155	76
50%	0.915	0.744	0.781	0.898	414	42
30%	0.915	0.744	0.605	0.953	546	25

Supplementary Table 8. Clinical utility at the deployed operating point across prevalences. Estimated at the fixed deployed operating point (sensitivity 0.915 and specificity 0.744 are properties of the model and therefore constant across prevalences; positive and negative predictive values derived by Bayes' theorem and clinical-impact counts expressed per 1,000 scans). The 89.4% row reflects the held-out test set prevalence, with the 50% and 30% rows representing lower-prevalence (more surveillance-realistic) settings.

Prevalence	PPV	NPV
10%	0.284	0.988
20%	0.472	0.972
50%	0.781	0.898
80%	0.935	0.687
89.4% (test)	0.968	0.510

Supplementary Table 9. Positive and negative predictive value as a function of disease prevalence. Derived by Bayes' theorem at the fixed deployed operating point (sensitivity 0.915, specificity 0.744). Negative predictive value (NPV) is limited at the high test-set prevalence (89.4%) but rises sharply at lower, surveillance-realistic prevalences, whereas positive predictive value (PPV) shows the converse pattern.

Set	n (GT+)	BA (95% CI)	Sensitivity	Specificity	Precision	F1	Voxel Dice (mean ± SD)
Full test set (N=1,109)	1,109 (992)	0.830 (0.791–0.872)	0.915	0.744	0.968	0.941	0.574 ± 0.319
NHNN-excluded external (n=712)	712 (642)	0.854 (0.798–0.900)	0.936	0.771	0.974	0.955	0.656 ± 0.291

Supplementary Table 10. Patient-level performance with and without the internal NHNN cohort. Patient-level nnU-Net metrics on the full held-out test set and on the external-only subset after excluding all internal NHNN cases. Balanced accuracy is higher on the external-only data than on the full set, bounding any concern about model-assisted-label circularity in the model's favour. Bootstrap 95% CIs (seed 42, 1,000 resamples); voxel Dice computed on ground-truth-positive cases only.

References

1. Ruffle JK, Mohinta S, Pombo G, et al. Brain tumour genetic network signatures of survival. *Brain* 2023; **146**(11): 4736–54.

2. Baid U, Ghodasara S, Bilello M, et al. The RSNA-ASNR-MICCAI BraTS 2021 Benchmark on Brain Tumor Segmentation and Radiogenomic Classification. *arXiv* 2021: arXiv:2107.02314.

3. van der Voort SR, Incekara F, Wijnenga MMJ, et al. The Erasmus Glioma Database (EGD): Structural MRI scans, WHO 2016 subtypes, and segmentations of 774 patients with glioma. *Data Brief* 2021; **37**: 107191.

4. Bakas S, Sako C, Akbari H, et al. The University of Pennsylvania glioblastoma (UPenn-GBM) cohort: advanced MRI, clinical, genomics, & radiomics. *Scientific Data* 2022; **9**(1): 453.

5. Calabrese E, Villanueva-Meyer JE, Rudie JD, et al. The University of California San Francisco Preoperative Diffuse Glioma MRI Dataset. *Radiol Artif Intell* 2022; **4**(6): e220058.

6. Correia de Verdier M, Saluja R, Gagnon L, et al. The 2024 Brain Tumor Segmentation (BraTS) Challenge: Glioma Segmentation on Post-treatment MRI. *arXiv e-prints* 2024: arXiv:2405.18368.

7. Moawad AW, Janas A, Baid U, et al. The Brain Tumor Segmentation - Metastases (BraTS-METS) Challenge 2023: Brain Metastasis Segmentation on Pre-treatment MRI. *ArXiv* 2024: arXiv:2306.00838v3.

8. Fathi Kazerooni A, Khalili N, Liu X, et al. BraTS-PEDs: Results of the Multi-Consortium International Pediatric Brain Tumor Segmentation Challenge 2023. *arXiv e-prints* 2024: arXiv:2407.08855.

9. LaBella D, Khanna O, McBurney-Lin S, et al. A multi-institutional meningioma MRI dataset for automated multi-sequence image segmentation. *Sci Data* 2024; **11**(1): 496.

10. Adewole M, Rudie JD, Gbadamosi A, et al. The Brain Tumor Segmentation (BraTS) Challenge 2023: Glioma Segmentation in Sub-Saharan Africa Patient Population (BraTS-Africa). *Neurooncol Adv* 2023; **8**(1): vdag082.

11. Kanber B, Ruffle J, Cardoso J, Ourselin S, Ciccarelli O. Neurosense: deep sensing of full or near-full coverage head/brain scans in human magnetic resonance imaging. *Neuroinformatics* 2020; **18**(2): 333-6.

12. Brudfors M, Balbastre Y, Nachev P, Ashburner J. MRI Super-Resolution Using Multi-channel Total Variation. *Medical Image Understanding and Analysis* 2018: 217-28.

13. Isensee F, Schell M, Pflueger I, et al. Automated brain extraction of multisequence MRI using artificial neural networks. *Human Brain Mapping* 2019; **40**(17): 4952-64.

14. Ruffle J, K., Mohinta S, Gray R, Hyare H, Nachev P. Brain tumour segmentation with incomplete imaging data. *Brain Commun* 2023; **5**(2).

15. Nachev P, Coulthard E, Jager HR, Kennard C, Husain M. Enantiomorphic normalization of focally lesioned brains. *Neuroimage* 2008; **39**(3): 1215-26.

16. Ashburner J, Friston KJ. Nonlinear spatial normalization using basis functions. *Hum Brain Mapp* 1999; **7**(4): 254-66.

17. Isensee F, Jaeger PF, Kohl SAA, Petersen J, Maier-Hein KH. nnU-Net: a self-configuring method for deep learning-based biomedical image segmentation. *Nat Methods* 2021; **18**(2): 203-11.

18. Myronenko A. 3D MRI brain tumor segmentation using autoencoder regularization. *arXiv* 2018: arXiv:1810.11654.

19. Hatamizadeh A, Nath V, Tang Y, Yang D, Roth H, Xu D. Swin UNETR: Swin Transformers for Semantic Segmentation of Brain Tumors in MRI Images. *arXiv* 2022: arXiv:2201.01266.

20. Hatamizadeh A, Tang Y, Nath V, et al. UNETR: Transformers for 3D Medical Image Segmentation. *arXiv* 2021.

21. Isensee F, Wald T, Ulrich C, et al. nnU-Net Revisited: A Call for Rigorous Validation in 3D Medical Image Segmentation. *arXiv e-prints* 2024: arXiv:2404.09556.

22. Ruffle JK, Gray RJ, Mohinta S, et al. Computational limits to the legibility of the imaged human brain. *NeuroImage* 2024: 120600.

23. He H, Garcia EA. Learning from Imbalanced Data. *IEEE Transactions on Knowledge and Data Engineering* 2009; **21**(9): 1263-84.

24. Brodersen KH, Ong CS, Stephan KE, Buhmann JM. The Balanced Accuracy and Its Posterior Distribution. *2010 20th International Conference on Pattern Recognition* 2010: 3121-4.

25. Ruffle JK, Mohinta S, Foulon C, et al. Fairboard: a quantitative framework for equity assessment of healthcare models 2026. https://ui.adsabs.harvard.edu/abs/2026arXiv260409656R (accessed March 01, 2026).

26. Hunter JD. Matplotlib: A 2D Graphics Environment. *Computing in Science & Engineering* 2007; **9**(3): 90-5.

27. The-MONAI-Consortium. Project MONAI. *Zenodo* 2020.

28. Brett, Matthew, Markiewicz, Hanke C. nipy/nibabel: 3.2.1 (Version 3.2.1). *Zenodo* 2020.

29. Nilearn-contributors. nilearn. 2024. https://github.com/nilearn/nilearn.

30. Harris CR, Millman KJ, van der Walt SJ, et al. Array programming with NumPy. *Nature* 2020; **585**(7825): 357-62.

31. Reback J, McKinney W, jbrockmendel. pandas-dev/pandas: Pandas 1.0.3 (Version v1.0.3). *Zenodo* 2020.

32. Paszke A, Gross S, Massa F, et al. PyTorch: An Imperative Style, High-Performance Deep Learning Library. *NeurIPS* 2019.

33. Waskom M, Seaborn-Development-Team. seaborn. *Zenodo* 2020.

34. Pedregosa F, Varoquaux G, Gramfort A. Scikit-learn: Machine Learning in Python. *Journal of Machine Learning Research* 2011; **12**: 2825-30.
